# Current progress in research on ocular injury caused by exposure to vesicants

**DOI:** 10.1016/j.preteyeres.2025.101413

**Published:** 2025-11-12

**Authors:** Marina Gorbatyuk, Nishant R. Sinha, Rajnish Kumar, Assylbek Zhylkibayev, Mohammad Athar, McNutt Patrick, Rajiv R. Mohan

**Affiliations:** a Wake Forest University Medical School, Department of Biochemistry, United States; b Wake Forest University Medical School, Institute for Regenerative Medicine, United States; c Wake Forest University Medical School, Department of Microbiology and Immunology, United States; d Harry S. Truman Memorial Veterans’ Hospital, Columbia, MO, United States; e University of Missouri College of Veterinary Medicine and School of Medicine, Departments of Ophthalmology, United States; f University of Alabama at Birmingham School of Medicine, Department of Dermatology, United States

## Abstract

Vesicants, powerful chemical weapons of mass destruction, are agents that cause blistering of the skin and blindness upon accidental exposure or during warfare. In addition, their exposure induces a wide range of other symptoms, including those affecting the respiratory tract, digestive system, skin, and ocular tissues. The ocular tissue can be exposed through both direct and indirect routes, such as direct contact with the affected corneal tissue and systemic blood circulation. Investigating eye injuries caused by vesicants is a critical and growing field of research in ophthalmology and ocular toxicology. In this review, we present the current status of the research, covering significant advances made in the study of vesicant-induced corneal and retinal injuries. We also provide general information on vesicants and discuss animal models used to investigate the molecular mechanisms of vesicant exposure and to develop medical countermeasures. Finally, we identify gaps in the current knowledge of the molecular mechanisms of vesicant action and highlight future directions for this emerging field.

## 1. Introduction

Vesicants are a class of chemical warfare agents that cause severe skin, eye, and mucosal irritation and blistering upon exposure. The most toxic forms of vesicants include compounds such as sulfur mustard (SM), nitrogen mustard (NM), and lewisite, which damage tissues through alkylation and oxidative mechanisms, resulting in inflammation, cellular death, and chronic injury. Vesicants first came into widespread use during World War I, with the deployment of SM by German forces starting with the Battle of Ypres in Belgium in July 1917. SM effectiveness as a chemical weapon derives from multiple physicochemical properties, including long environmental persistence, rapid toxicity at low vapor doses, asymptomatic exposure, and the development of incapacitating morbidities after a short latent period (Balali-Mood and Hefazi, 2005; Satkin et al., 2017; Wattana and Bey, 2009). The extraordinary effectiveness of SM as a battlefield weapon led to its widespread use throughout the remainder of World War I, producing 77 % of all chemical injuries and 1.2 million casualties, with the majority of survivors suffering permanent effects, including respiratory complications, blindness, scarring, and other chronic health issues (Fitzgerald, 2008; Hughes, 1945; McNutt et al., 2020a). Despite widespread condemnation and subsequent international agreements, including the Geneva Protocol of 1925 prohibiting chemical warfare, SM continued to be used in later conflicts, most notably by Italy against Ethiopian forces during the Second Italo-Ethiopian War (1935–1936); Japan against Chinese forces in the Second Sino-Japanese War (1937–1945); and Iraq during the Iran-Iraq War (1980–1988), where Iraqi forces systematically employed sulfur mustard against Iranian troops and Kurdish populations. Sporadic instances of sulfur mustard use have persisted into the 21st century, including reports of deployment by the Islamic State (ISIS) and the Syrian government in conflicts in Iraq and Syria around 2015–2016 (Singh et al., 2023).

Although there is no confirmed battlefield deployment of lewisite, it is believed that Japan may have used the agent against China between 1937 and 1944. One suspected incident in the Yangtze Valley involved a combination of chemical agents—including lewisite, mustard gas, phosgene, hydrogen cyanide, and various tear gases—which reportedly led to the deaths of more than 3,500 Chinese civilians. Unlike sulfur mustard, lewisite causes immediate pain and involuntary blinking, especially when released in aerosol form (Mann et al., 1946).

**Fig. 1.**
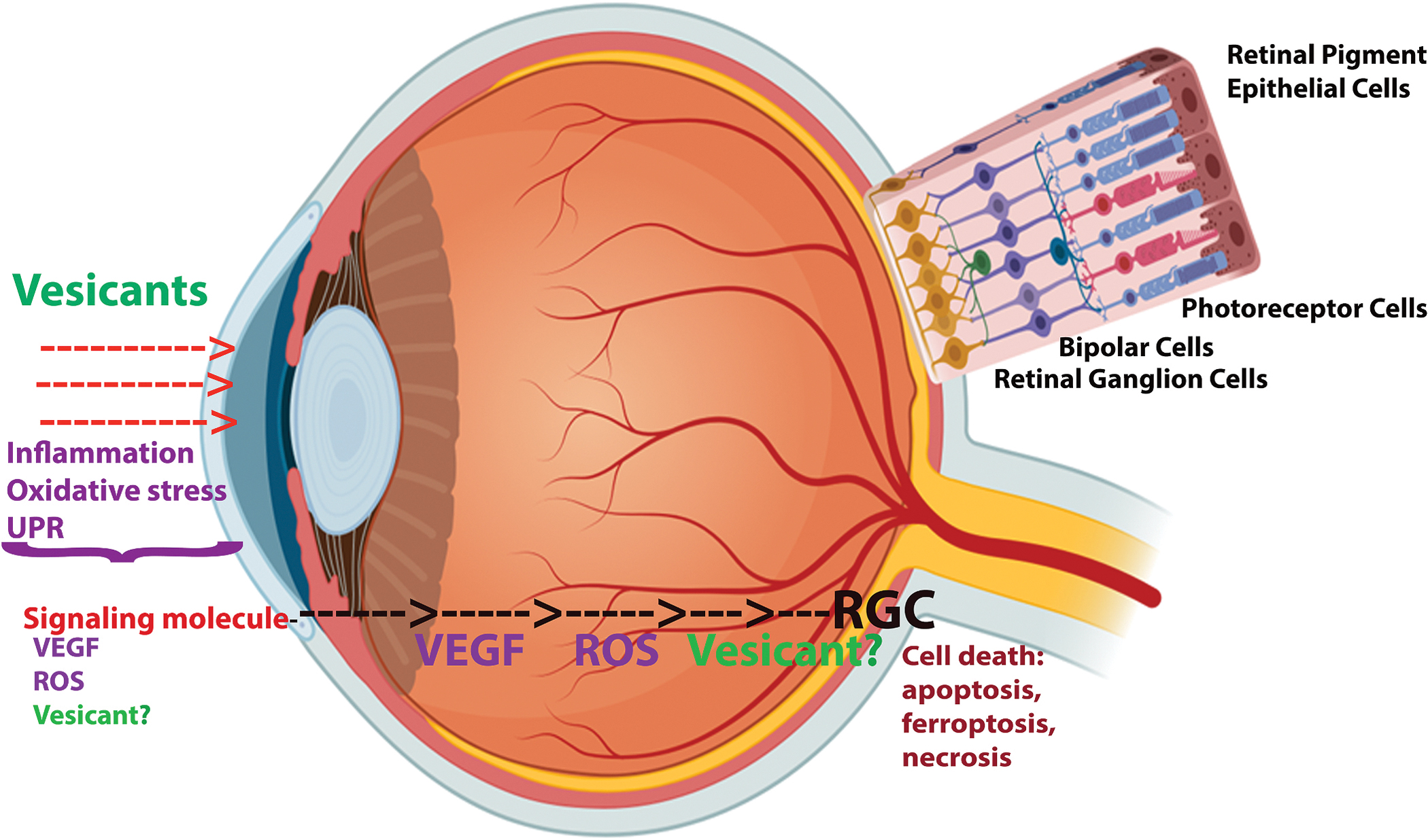
Direct ocular exposure to vesicants leads to both corneal and retinal damage. In the cornea, key cellular signaling pathways—including inflammation, oxidative stress with the generation of reactive oxygen species (ROS), and the unfolded protein response (UPR)—are rapidly activated. These stress responses can lead to the release of signaling molecules such as VEGF and ROS, which may diffuse posteriorly and contribute to secondary retinal injury. Additionally, vesicants may directly reach the retina by diffusing through the vitreous humor, causing retinal cell death via apoptosis, ferroptosis, and/or necrosis or through blood circulation. Thus, in cases of systemic exposure, retinal injury can occur via two primary mechanisms: (1) direct transmission following corneal exposure, and (2) indirect exposure through ocular blood circulation. Both routes have the potential to disrupt retinal cell homeostasis and function.

Vesicants can be dispersed as liquids, gases, or aerosols. The development of clinical symptoms varies widely, depending on the specific vesicant and the level of exposure. Respiratory effects often include coughing, shortness of breath, nasal discharge, sneezing, hoarseness, nosebleeds, and sinus discomfort. Mild exposures generally result in symptom onset within 12–24 h, whereas severe exposures may cause symptoms to appear more rapidly, typically within 2–4 h (Malaviya et al., 2020). Vesicant exposure can also impact the digestive system, leading to abdominal discomfort, diarrhea, fever, nausea, and vomiting (Dacre and Goldman, 1996). Skin manifestations frequently include blister formation, redness, pain, and swelling (Tewari-Singh et al., 2017b). However, the eyes are particularly vulnerable to vesicant injury due to their external exposure, the high metabolic rate of corneal epithelial cells, the tear film’s absorptive capacity, and the limited regenerative potential of ocular tissues (McNutt, 2023). Common ocular signs and symptoms of vesicant exposure include pain, swelling, excessive tearing, conjunctivitis, corneal lesions, ulcers, and opacity (Goswami et al., 2016a). Importantly, ocular exposure can occur both directly through contact with vesicants and indirectly via systemic blood circulation (Fig. 1). The investigation of vesicant-induced eye injuries is critically important, as these agents pose a substantial risk of large-scale, severe and long-lasting ocular damage that leads to impaired or lost vision. The demonstrated efficacy of vesicants as chemical munitions raises the likelihood of future use in warfare or terrorist activities, underscoring a critical public health threat and emphasizing the importance of research aimed at elucidating the mechanisms of injury, identifying therapeutic targets, and validating effective medical countermeasures to minimize harm (Araj et al., 2022).

A substantial body of evidence in the literature highlights intensive research conducted on corneal tissue exposed to various vesicants, while only initial steps have been taken toward understanding the consequences of direct ocular exposure on the retina. In this review, we primarily focus on corneal and retinal tissues in the context of ocular exposure to vesicants.This review article provides up-to-date insights into the current status of research, with particular emphasis on agents whose mechanisms of action are well characterized. We highlight the use of both *in vitro* and *in vivo* models that have been instrumental in elucidating the mechanisms of ocular tissue injury and the associated pathophysiological responses. Furthermore, emerging directions in the development of novel medical countermeasures are explored, including pharmacological interventions and gene therapy approaches aimed at mitigating acute damage and promoting long-term retinal recovery.

## 2. Toxic mechanisms of action of vesicant agents

**Fig. 2.**
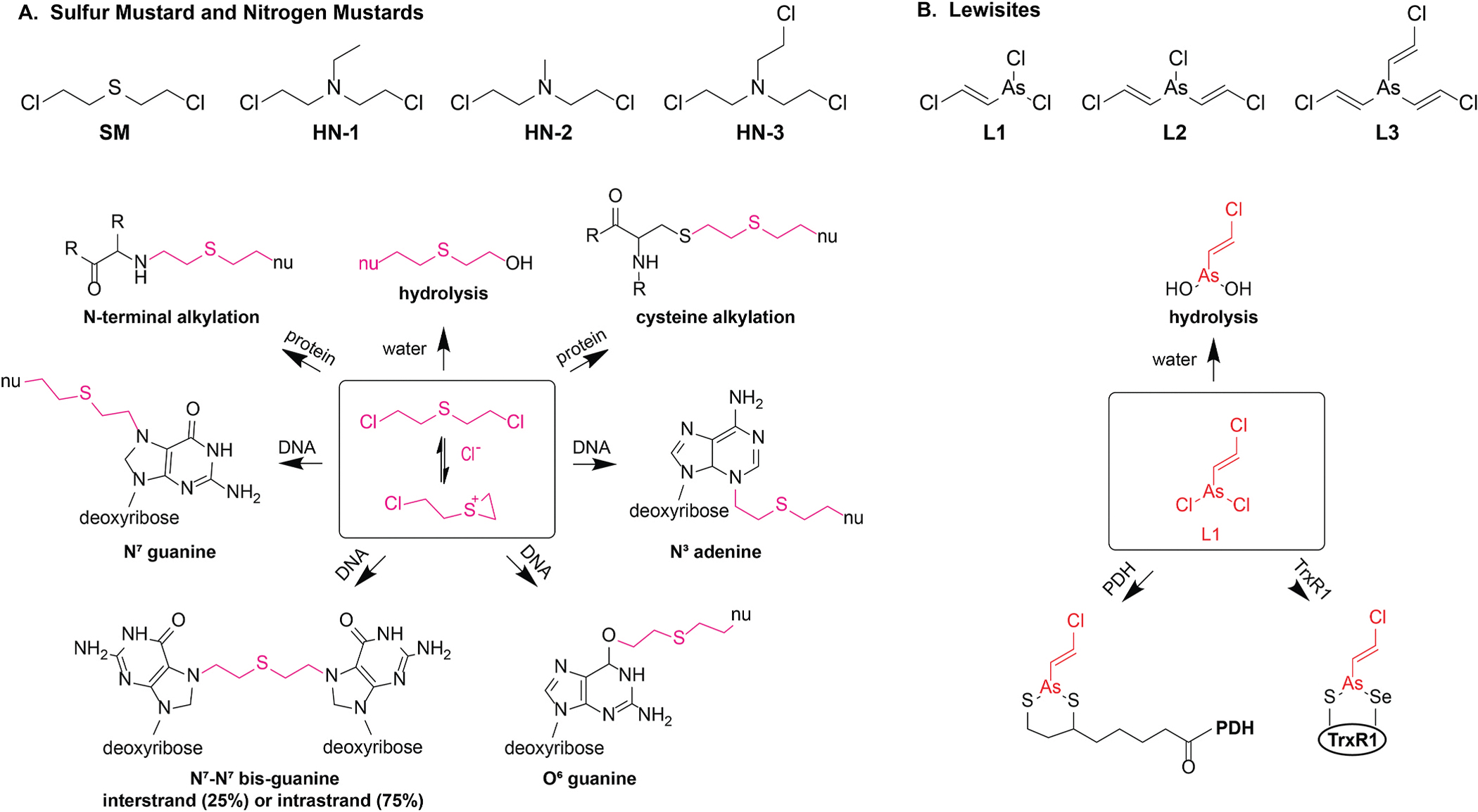
Structure and representative molecular targets of Schedule 1 vesicants. (**A**)Top: structures of sulfur mustard and nitrogen mustards. Bottom: schematic of intramolecular cyclization to three-membered onium ring for sulfur mustard and subsequent fates in physiological media. Hydrolysis by water yields non-alkylating products, whereas nucleophilic attack on the β-carbon yields covalent alkylation of DNA bases and protein side chains. The schematics depict reaction classes and targets rather than exhaustive stoichiometries. Reactions are equivalent for nitrogen mustards. “nu” indicates hydrolysis or alkylation of additional targets by remaining 2-chloroethyl arm(s). (**B**) Top: structure of lewisites. Bottom: schematic of As(III) coordination chemistry. In aqueous biological media, chloride ligands are substituted stepwise by cellular thiolates and selenolates. Shown are representative targets: the vicinal Cys–Cys dithiol on the E2 dihydrolipoamide acetyltransferase component of the pyruvate dehydrogenase (PDH) complex, which forms a stable bidentate chelate that arrests acyl transfer, and the Cys–Sec redox center of thioredoxin reductase 1 (TrxR1), whose coordination inhibits electron transfer and shifts the cellular redox setpoint. Hydrolysis pathways are shown for completeness but are kinetically minor relative to thiol or selenol substitution.

Despite their divergent chemistries, sulfur mustard (SM; bis(2-chloroethyl) sulfide), nitrogen mustards (HN-1, bis(2-chloroethyl) ethylamine; HN-2, bis(2-chloroethyl)methylamine; HN-3, tris(2-chloroethyl)amine), and lewisites (L1, 2-chlorovinyldichloroarsine; L2, bis(2-chlorovinyl)chloroarsine; L3, tris(2-chlorovinyl)arsine) produce convergent toxicological effects central to their Schedule 1 classification (Fig. 2) (Pechura and Rall, 1993; McNutt et al., 2020a). These vesicants are environmentally persistent and pose vapor and liquid hazards at ambient temperature. As small and lipophilic molecules, they readily partition into lipid-aqueous interfaces such as tear film or mucus, and are readily absorbed across the skin, ocular surface, and airway. The ocular surface and respiratory mucosa have particularly low exposure thresholds because of thin epidermal barriers, high surface area, and biological interfaces that favor absorption. At the point of exposure, these vesicants generate short-lived reactive intermediates that covalently modify DNA, proteins, and lipids, producing acute molecular toxicities that activate secondary injury responses. Dose-response follows concentration-time scaling, so short-duration, high-dose exposures, and long-duration, low-dose exposures can have similar outcomes (Arroyo et al., 2004). Exposure to these agents causes acute compromise of epithelial integrity with sloughing and vesication and can lead to blindness, respiratory failure and death, albeit through distinct chemistries and toxicokinetics.

Vesicant injury trajectory is agent-specific and is governed by the half-life of the reactive intermediate, target selectivity, and pharmacokinetic properties such as absorption and distribution (Pechura and Rall, 1993). The local microenvironment strongly influences molecule toxicology (Hamlin et al., 2018). Phase behavior at lipid aqueous boundaries, the availability of free water, macromolecular crowding, and local electrostatics sculpt the molecular interactions and contribute to tissue-specific responses across skin, eye, and airway. Cellular responses to molecular toxicology converge on key pathways, including bioenergetic disruption through depletion of NAD^+^ and ATP, redox and thiol disequilibrium, and proteostasis pressure within the endoplasmic reticulum and chaperone systems (Beigi Harchegani et al., 2019; Goldman and Dacre, 1989; Jan et al., 2010; Jan et al., 2014; Kehe et al., 2009; Li et al., 2016b; Mangerich et al., 2016; Ramos et al., 2023; Ruszkiewicz et al., 2020). These proximal events exacerbate sterile inflammatory responses that amplify tissue injury. Moist interfaces, such as mucous or tear film, promote capture and absorption of vesicant vapors by underlying ocular or airway tissues. Clinical symptoms of mustard exposure manifest following a latent period of 12–24 h, during which cells detect and process alkylation events (Balali-Mood and Hefazi, 2005; Dacre and Goldman, 1996; Guild et al., 1941). This clinically latent period increases the chance of extended exposure and can delay timely decontamination or nucleophile scavenging. In contrast, lewisites usually cause immediate pain and irritation that signals exposure and can trigger rapid decontamination and dithiol chelation (Goldman and Dacre, 1989). Outside these early windows, interventions must shift from preventing or reversing molecular toxicology to stabilizing bioenergetics, limiting secondary oxidative and electrophilic damage, preserving barrier function, controlling inflammation and promoting regeneration.

Mustards toxicity originates with intramolecular cyclization (McNutt, 2023). Under physiological conditions, the intramolecular sulfur or nitrogen atom displaces a chloride ion from one of the chloroethyl side chains, forming a highly strained, three-membered cyclic onium ion (episulfonium for SM and aziridinium for HN) (Fig. 2). SM, HN-1, and HN-2 are bifunctional and can form onium ions with each 2-chloroethyl arm in sequence, whereas HN-3 is trifunctional. In neat mustard, the absence of a stabilizing polar solvent and of a nucleophile sink favors reversion via chloride capture to the parent compound (Hemstrom et al., 2020; Prasad and Singh, 2004). However, in physiological media the onium ion is highly susceptible to nucleophilic attack by water, DNA, RNA or protein side chains (typically Cys or His) (McNutt, 2023). Hydrolysis by water results in the production of a non-toxic thiodiglycol, whereas attack by nucleic acids or protein results in covalent alkylation. The balance between hydrolysis and alkylation depends on microenvironmental factors (Hamlin et al., 2018; Papirmeister et al., 1991a). Alkylation predominates in poorly hydrated niches such as lipid layers, dehydrated interfaces, and protein pockets, whereas hydrolysis is favored in bulk aqueous phases. Although the cytosol and nucleoplasm are near bulk water and therefore support strong hydrolytic competition, nucleophile density and electrostatic focusing (primarily attraction of cationic onium ions to the polyanionic DNA backbone and the high effective molarity of nucleophilic sites in chromatin and protein pockets) bias reactions toward alkylation.

Cellular DNA is a key toxicological target of mustard alkylation, with the production of multiple forms of genotoxic lesions (Shahin et al., 2001). Following an alkylation event on DNA, the second (or third) onium ion can react with: 1) DNA, producing interstrand or intrastrand crosslinks, 2) protein, generating DNA:protein crosslinks, or 3) water, yielding a mono-alkylated base (Fig. 2). Mass spectrometry analyses of DNA from SM-exposed human cells reveal alkylation at N^7^ guanine > N^3^ adenine ≫ O^6^ guanine, with 75–90 % mono-alkylation events and 10–25 % cross-linked DNA (Fidder et al., 1994; Kehe et al., 2008; Kircher and Brendel, 1983; Lawley and Brookes, 1967; Ludlum et al., 1986, 1994; Mattes et al., 1986; Walker, 1971; Wang et al., 2015; Zubel et al., 2019). Intrastrand DNA crosslinks and interstrand DNA crosslinks (ICL) are observed in an approximate 3:1 ratio. DNA:protein crosslinks involving hundreds of proteins have also been reported (Groehler et al., 2016; Loeber et al., 2009). These highly toxic crosslinks impede replication and must be cleared through proteolysis-coupled repair, such as via SPRTN and the proteasome (Stingele et al., 2017). Finally, mitochondrial DNA can also be alkylated by mustards, damaging membrane potential, increasing reactive oxygen species and exacerbating metabolic stress (Cline, 2012; Gould et al., 2009; Shahin et al., 2001).

In general, the production of alkylated DNA species activates multiple pathological responses (Mattes et al., 1986; McNutt, 2023). The deleterious impact of DNA damage responses on cytotoxicity is illustrated in experiments with repair-deficient cells, which found that cells deficient in base excision repair (BER) are more sensitive to the monoalkylating half-mustard 2-chloroethyl ethyl sulfide (CEES) than SM, whereas cells deficient in non-homologous end-joining and homologous recombination are more sensitive to SM than CEES (Jowsey et al., 2012). The production of ICL represents a particularly critical toxicological outcome. Whereas limited quantities of DNA monoalkylation and intrastrand crosslinks can be repaired using BER and nucleotide excision repair (NER) pathways (Clauson et al., 2013; Jowsey et al., 2012; Lopez-Martinez et al., 2016; Mol et al., 1993; O’Donovan et al., 1994; Richterova et al., 2018), ICLs must be repaired by the Fanconi anemia (FA)-dependent repair system. FA is a complicated pathway, involving FANCD2 and FANCI monoubiquitination, recruitment of nucleases to create a single-strand gap, engagement of translesion synthesis polymerases to traverse the ICL, and resolution of the resulting double strand breaks by homologous recombination (Dronkert and Kanaar, 2001; Jung et al., 2020; Kottemann and Smogorzewska, 2013). This pathway is complex and inefficient, and failure at any step yields chromosome breakage and catastrophic mitoses (Batal et al., 2014; Dronkert and Kanaar, 2001; Semlow and Walter, 2021). Because very few ICLs can trigger checkpoint activation and loss of viability, interstrand crosslinks are considered a primary driver of cytotoxicity, particularly at low mustard doses (Matijasevic et al., 2001). Alternatively, high densities of DNA alkylation can trigger acute metabolic consequences through activation of the central DNA damage repair regulator Poly (ADP-ribose) polymerase-1 (PARP-1) (Liu et al., 2016; Mangerich et al., 2016). PARP-1 responds rapidly to DNA damage by adding branched ADP-ribose moieties to chromatin-bound proteins to recruit DNA repair enzymes (van Beek et al., 2021). PARP-1 activation is energetically expensive, and overstimulation of PARP-1 leads to NAD^+^ exhaustion and ATP depletion, thereby shifting cell death pathways from apoptosis (e.g. due to ICL formation) to PARP-mediated parthanatos (Murata et al., 2019; van Beek et al., 2021). PARP inhibition or NAD^+^ supplementation attenuates mustard-induced NAD^+^/ATP loss and cell death *in vitro* (Liu et al., 2016; Mangerich et al., 2016), underscoring PARP-driven NAD^+^ depletion as a key early event rather than a downstream epiphenomenon.

Although less studied than genotoxic effects, protein alkylation also has direct consequences to cellular injury by mustards. Proteome-wide alkylation perturbs catalytic throughput in glycolytic and redox enzymes, loads chaperone systems, and destabilizes cytoskeletal architecture (Borna et al., 2019; Jan et al., 2010; Jan et al., 2014). These post-translational changes engage the unfolded protein response (UPR), endoplasmic reticulum stress signaling, and autophagic and lysosomal compensation prior to transcriptional activation by genotoxic injuries (Borna et al., 2019; Kehe et al., 2009; Owens et al., 2025). DNA:histone crosslinks add a mechanical barrier to replication and repair that contribute to replication failure responses (Groehler et al., 2016; Loeber et al., 2009). Proteomic surveys identify creatine kinase and alpha 1 antitrypsin among intracellular alkylation targets (Lüling et al., 2021; Steinritz et al., 2021), which implies acute compromise of energy buffering and protease inhibition. In basal epithelial cells, alkylation of beta actin, cytokeratins, and actin regulatory proteins impair polymerization dynamics and focal adhesion turnover, trigger hemidesmosome disassembly, and promote epithelial detachment (Borna et al., 2019; Kehe et al., 2009; Owens et al., 2025). These direct post-translational modifications precede transcription-based inflammatory responses. However, due to the general preference of mustards for hard nucleophiles, protein-level cellular effects in response to alkylation of soft nucleophile protein side chains are more likely to emerge at higher doses than genotoxic lesions, and therefore the pathological importance of protein alkylation remains uncertain.

At the biochemical level, early cellular responses to mustard exposure are dominated by DNA damage recognition and metabolic dysfunction (Beigi Harchegani et al., 2019; Goldman and Dacre, 1989; Jan et al., 2010; Jan et al., 2014; Kehe et al., 2009; Li et al., 2016b; Mangerich et al., 2016; Ramos et al., 2023; Ruszkiewicz et al., 2020). Reactive oxygen species (ROS) arise from impaired electron transport, calcium dysregulation, and glutathione (GSH) depletion that reflects thiol alkylation and increased antioxidant demand (Beigi Harchegani et al., 2019). Lipid peroxidation increases and yields α,β-unsaturated aldehydes such as 4-HNE, which propagate secondary protein modification through Michael addition chemistry (Ruszkiewicz et al., 2020). These oxidative and electrophilic stresses integrate with PARP-driven energy failure to impair proteostasis, promote protein aggregation, and shift the UPR from adaptive to pro-apoptotic programs, such as induction of C/EBP homologous protein (CHOP) (Kehe et al., 2009; Owens et al., 2025). In animal models, markers of endoplasmic reticulum stress including GRP78, BiP, and CHOP increase within 24 h after mustard exposure (Chang et al., 2013). This pattern is consistent with an initial adaptive response that is rapidly overwhelmed when alkylation burden is high or ATP is low. At modest exposure, cells mobilize thiol redox switches such as thioredoxin, peroxiredoxin, and glutaredoxin systems and transiently suppress translation. At higher exposure, mixed cell death modes often coexist within the same tissue (Bosholm et al., 2023; Dabrowska et al., 1996; Gao and Yu, 2025; Kehe et al., 2009; Liu et al., 2016; Owens et al., 2025; Sinha et al., 2025a; Zhylkibayev et al., 2024).

Lewisite toxicity begins with coordination chemistry at trivalent arsenic. In biological media the arsenic center behaves as a soft Lewis acid that prefers soft nucleophiles, especially thiolates and selenolates (Goldman and Dacre, 1989). The dominant reactions are thiol or selenol substitutions rather than slow hydrolysis. The first events are ligand-exchange reactions, in which the Lewisite chlorides are sequentially replaced by cellular nucleophiles. Monodentate thiolates form As-S coordinate covalent bonds to give mixed chloro-thiolate species, and rapid subsequent substitution yields bis- and tris-thiolate complexes. This preference explains the strong reactivity toward the reduced form of lipoamide on 2-oxoacid dehydrogenase complexes. Specifically, lipoamide is a cofactor covalently attached to the E2 subunit of pyruvate dehydrogenase (PDH) and α-ketoglutarate dehydrogenase (KDH) that cycles between oxidized (disulfide) and reduced (dithiol) forms during the TCA cycle. In its reduced form, lipoamide contains two free thiol (−SH) groups that are geometrically ideal for lewisite coordination, immobilizing the lipoyl cofactor and blocking acyl transfer to coenzyme A (Fig. 2). This mechanism is central to lewisite’s systemic toxicity and explains why thiol-rich cofactors like lipoamide are among the earliest and most sensitive intracellular targets. Similarly, selenocysteine in thioredoxin reductase and related selenoenzymes presents a highly nucleophilic chalcogen that forms very stable As-Se interactions (Ingold and Conrad, 2018). The TrxR1 S-Se redox center provides a high-affinity, bidentate binding site that is inhibited by lewisite within minutes, oxidizing thioredoxin, inhibiting electron transfer through the FAD and shifting the redox setpoint toward oxidative stress (Park, 2020; Wright and O’Donoghue, 2024). These interactions result in the immediate inhibition of lipoamide dependent dehydrogenases and the early inhibition of redox maintenance enzymes, which collectively produce an ATP deficit, increase reactive oxygen species, and disrupt ionic homeostasis within minutes, without involving DNA damage or transcriptional changes (Goldman and Dacre, 1989; Ruszkiewicz et al., 2020). The mechanistic progression from lewisite coordination with sulfur or selenium-containing proteins to redox collapse and bioenergetic failure is central to the acute cellular effects of lewisite and is a key distinction from the delayed effects and toxic mechanisms of the mustards.

Additional coordination features of lewisite may contribute to cellular toxicity. First, As(III) can bind to clusters of cysteines in metal binding domains such as zinc finger motifs, producing misfolding and loss of function (Zhao et al., 2012; Zhou et al., 2014). While zinc finger disruption contributes to genotoxic synergy and transcriptional dysregulation, inhibition of lipoamide-containing enzymes (e.g. PDH, KDH) and selenoproteins like thioredoxin reductase has a more immediate and systemic impact on energy metabolism, redox balance, and cell viability. Because As(III) can accept up to three soft donors, it can bind vinical thiols both within and between proximal proteins, stabilizing inactive conformations and promoting intracellular aggregation (Andersson et al., 2021; Cholanians et al., 2016; Jacobson et al., 2012). Finally, As (III) coordination to cysteines on chemosensory proteins and channels (such as the TRP channels) provides a plausible mechanism for acute sensitive nociceptor activation (Goldman and Dacre, 1989; Steinritz et al., 2018), which supports observations of immediate pain and reflex blepharospasm or withdrawal upon exposure.

**Fig. 3.**
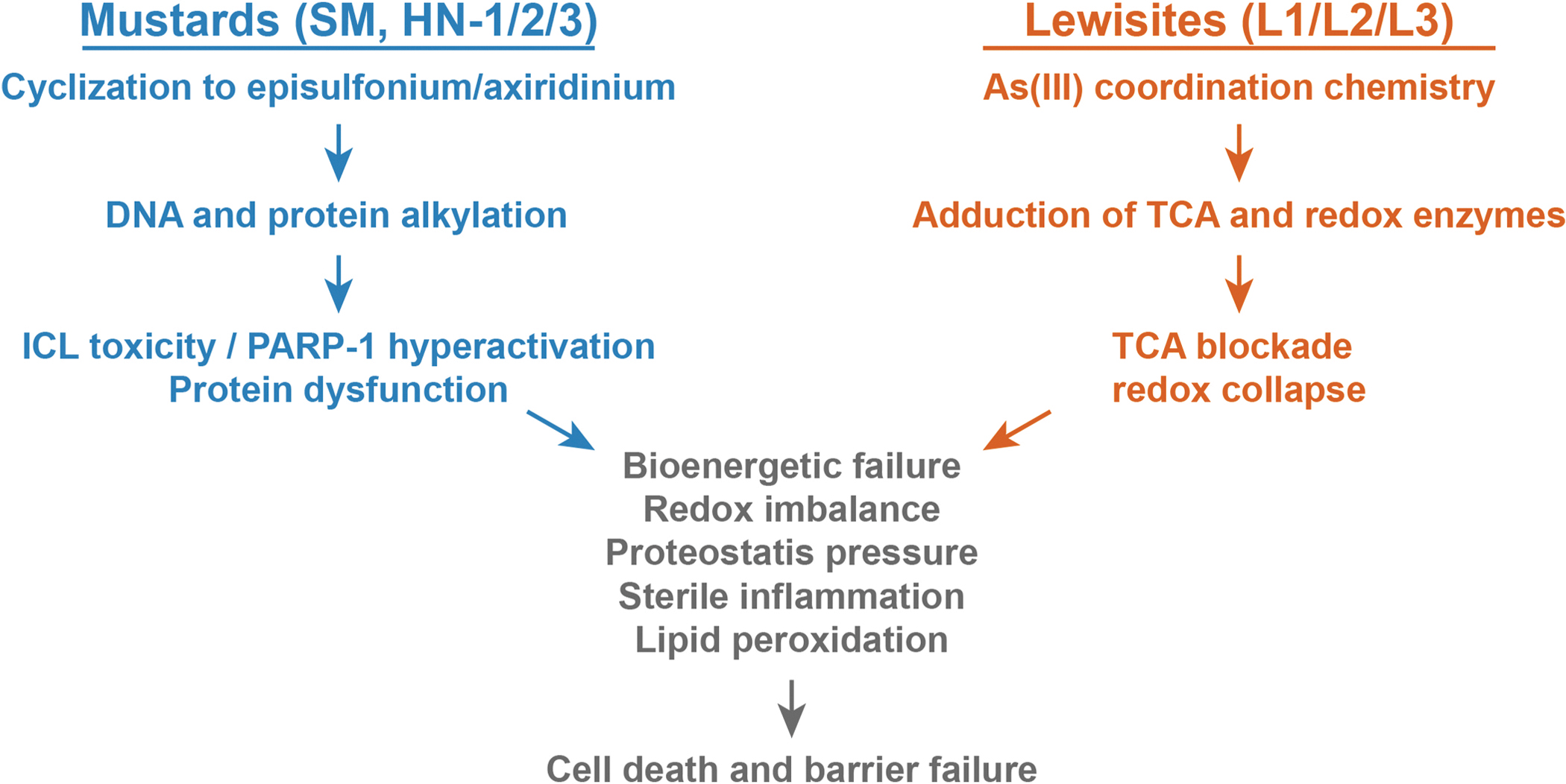
Overview of the toxic mechanisms of action for mustards and lewisites. Despite their distinct chemistry and generally non-overlapping molecular targets, the mustards and lewisites converge to produce cytotoxic effects of mustard alkylation and lewisite coordination converge to activate cell death pathways. (**Left**) Mustards cyclize to short-lived onium ions that undergo SN2 attack to yield DNA mono-adducts, interstrand (ICL) and intrastrand crosslinks, and DNA:protein crosslinks (DPC), with concurrent protein alkylation of enzymes and cytoskeletal elements. These primary lesions trigger checkpoint failure (ICL) pathways, overstimulate PARP-1, deplete NAD^+^, constrain glycolysis, and depress ATP. (**Right**) Lewisites coordinate with cellular thiolates and selenolates with preference for vicinal 1,2-dithiols and selenocysteine, targeting lipoyl domains on pyruvate and α-ketoglutarate dehydrogenase complexes and thioredoxin reductase. Coordination at these sites blocks substrate entry into the tricarboxylic acid cycle, collapses ATP supply, and shifts the cellular redox setpoint toward oxidative stress without a genotoxic latency. (**Center**) The vesicant families functionally converge to elicit a shared set of cytotoxic effects that trigger cell death and barrier failure.

Collectively, a comparison of the molecular and cellular responses to the mustards and lewisites illustrates a convergence on several cytopathological mechanisms (Fig. 3). Both vesicant families initiate injury at biological interfaces within minutes, drive early bioenergetic failure, elicit redox imbalance and proteostasis stress, and culminate in cell death and barrier breakdown with sterile inflammation that amplifies the cytotoxic lesion. In mustards, cyclization to short-lived onium ions followed by nucleophilic attack yields DNA monoalkylation, interstrand and intrastrand DNA crosslinks, and DNA:protein crosslinks, while parallel protein alkylation disrupts enzymes, cytoskeleton, and chaperone capacity. The aggregate toxicological load activates PARP-1, depletes NAD plus, depresses ATP, and pushes cells toward non-apoptotic modes of cell death at higher exposure levels, while the formation of interstrand crosslinks trigger checkpoint failure and initiates apoptotic cell death pathways. In lewisites, As(III) rapidly coordinates vicinal dithiols and selenols, inhibiting lipoamide-dependent dehydrogenases and thioredoxin reductase, which blocks substrate entry into the tricarboxylic acid cycle, collapses ATP supply, and shifts the redox setpoint toward oxidative stress without the latency of mustard-induced genotoxic responses. Across tissues, microenvironmental factors such as water availability, macromolecular crowding, and local electrostatics bias the first reactions and explain why thin interfaces like corneal epithelium and respiratory mucosa show low injury thresholds. In all cases, the secondary inflammatory response to cell death exacerbates edema, protease activity, and oxidative damage. These common cytopathological endpoints have practical implications for cell-based therapies: early actions to mitigate molecular toxicity have a narrow therapeutic window but could be definitive, whereas delayed treatments should manage organ-level fibrotic responses and promote effective tissue regeneration.

## 3. Ocular vesicant injury in humans from a clinical perspective

**Fig. 4.**
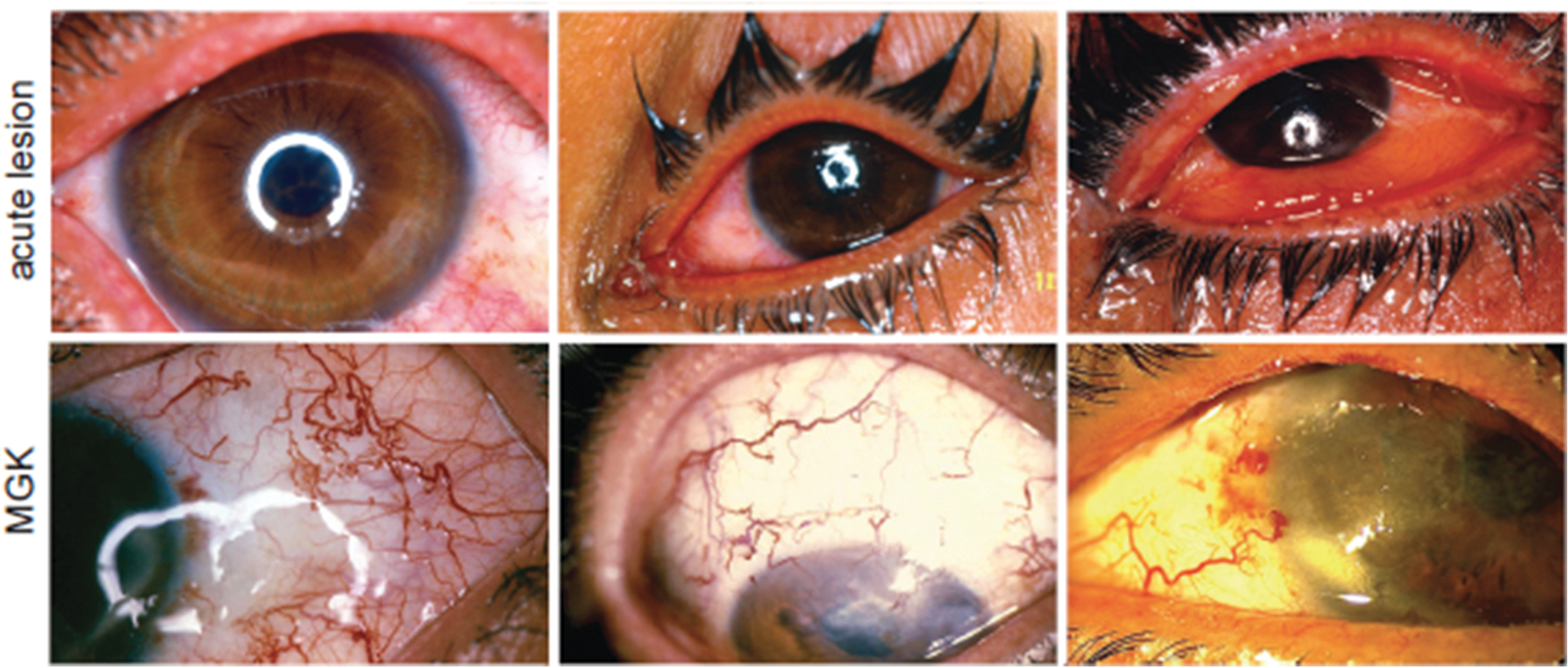
SM-induced pathophysiological effects on the eye. Representative images of human eyes demonstrating acute SM lesions (top) and MGK (bottom; modified from (Rajavi et al., 2017)). The acute severity is ranged from mild (left panel, conjunctival hyperemia, vascular dilation and no corneal involvement), moderate (center panel, mild signs plus corneal involvement and generalized edema) and severe (right panel, moderate signs plus corneal inflammation, corneal edema and corneal defects). The MGK images illustrate the development of irreversible pathologies such as neovascularization and corneal opacity (Republished with permission from the Experimental Eye Research journal).

Clinical data collected from battlefield exposures, low-dose clinical studies and accidental exposures at ammunition plants has produced robust concentration-time responses for ocular SM vapor exposures (Anderson, 1942; Balali-Mood and Hefazi, 2006; Guild et al., 1941; Hughes, 1945; Reed et al., 1918; Solberg et al., 1997; Uhde, 1946) (Fig. 4). The acute effects of SM injury typically emerge after a latent period that lasts from 2 to 24 h. SM vapor doses <60 min mg/m^3^ result in mild conjunctival injection, without corneal involvement, that resolves within 1–2 weeks (Guild et al., 1941; Hughes, 1945; Solberg et al., 1997). Vapor doses ranging from 60 to 200 min mg/m^3^ produce eyelid, conjunctival, and mild-to-moderate corneal lesions that required 2–6 weeks to resolve. Vapor doses exceeding 200 mg min/m^3^ cause severe corneal lesions that required 12 weeks or longer to resolve. Furthermore, corneas exposed to >200 mg min/m^3^ can develop a constellation of persistent pathologies known as mustard gas keratopathy (MGK) involving recurring corneal epithelial lesions, corneal neovascularization, opacity, and progressive corneal degeneration, which in turn cause persistent symptoms such as photophobia, ocular pain, blepharospasm, and, in severe cases, impaired or lost vision (Balali-Mood and Hefazi, 2006; Hughes, 1945; Javadi et al., 2005; Rajavi et al., 2017; Sidell et al., 1997; Solberg et al., 1997). MGK may arise shortly after the acute injury (chronic form) or develop after a subacute phase lasting 0.5–40 years (delayed-onset form). Delayed-onset MGK typically appears with the abrupt development of photophobia, tearing, and corneal and limbal lesions, with a steep increase in incidence between 17 and 25 years after exposure (Balali-Mood and Hefazi, 2006; Hughes, 1945; Javadi et al., 2005; Mann and Pullinger, 1942; Rajavi et al., 2017; Sidell et al., 1997; Solberg et al., 1997). In longitudinal studies, up to 35–95 % of survivors of severe SM ocular exposure developed symptoms of MGK, including photophobia, tearing, foreign body sensation, corneal neovascularization, and corneal erosions (Balali-Mood and Hefazi, 2006; Javadi et al., 2005; Solberg et al., 1997). Of these, approximately 1 % develop advanced corneal disease characterized by progressive corneal degeneration and impairment or loss of vision. Despite the temporal differences in clinical onset, chronic and delayed-onset forms of MGK have similar symptoms and may share a common pathophysiology, Recent clinical investigations have provided critical insights into the long-term effects of vesicant exposure on the ocular surface. Survivors of vesicant SM exposure often develop chronic ocular surface disorders, including persistent epithelial defects, dry eye disease, limbal stem cell deficiency, conjunctival goblet cell loss, and meibomian gland dysfunction (Soleimani et al., 2023). The other chronic symptoms were blepharitis, limbal ischemia, conjunctival and corneal neovascularization, and secondary degenerative changes such as lipid and amyloid deposition, corneal thinning, stromal haze, and surface irregularities (Javadi et al., 2005). These reports highlight the vulnerability of eye to delayed complications and warrant the development of effective medical countermeasures (MCMs) addressing both acute and chronic ocular surface dysfunction.

Exposure to a mixture of SM and lewisite in the 2003 accident in Qiqihar, northeast China, highlights the severity, spectrum, and delayed appearance of multiple clinical manifestations, including corneal damage and constricted vision (Isono et al., 2018). The 44 victims were poisoned by these CWAs. Six regular check-ups were conducted in the period between 2006 and 2014, and from 2008 onward, these survivors underwent neuropsychological and autonomic nervous function tests. The clinical manifestations in the exposed individuals included severe autonomic failure, such as hyperhidrosis and pollakiuria. Memory and visuospatial abilities were also affected in the survivors, suggesting that SM/lewisite exposure had significant adverse consequences on cognitive and autonomic nervous system functions in the brain. These studies demonstrated how easy it is to underestimate many ocular and neurological symptoms, which may emerge decades later or persist long-term. Indeed, the retrospective study with survivors of the SM terroristic attack conducted in 2017 reported significant reduction of retinal function in 40 severely intoxicated Iranian veterans (Shoeibi et al., 2017). The max A- and B-wave of the scotopic and photopic ERGs as well as implicit time of responses were significantly diminished in these individuals overall supporting the earlier conducted research detected central retinal vein occlusion, macular edema, diminished vision acuity (Shoeibi et al., 2016) and overexpression of VEGF-A in tears (Abbaszadeh et al., 2014) of the survivors of the IIW SM attack.

Overall, these published studies demonstrated that the clinical appearance of the corneal and retinal damage as a result of mentioned above VS exposures, depends on the type, dose, and duration of follow-up examinations.

## 4. *In vitro*, *ex vivo*, and *in vivo* models of ocular exposure to vesicants

### 4.1. Models of corneal injury

A variety of experimental models are used to uncover pathological mechanisms, evaluate pathophysiological processes, study functional outcomes, and develop therapeutic interventions for vesicant exposures. These models are broadly categorized into *in vitro, ex vivo*, and *in vivo* systems, each offering distinct advantages and limitations that make them indispensable for different stages of research. While *in vitro* models provide foundational insights at the molecular and cellular levels, *ex vivo* models bridge this gap by preserving tissue architecture and enabling detailed drug penetration studies. *In vivo* models, on the other hand, offer a holistic view of injury progression and therapeutic outcomes under conditions closely resembling clinical scenarios. Integrating these models enhances translational research by leveraging their respective strengths. For example, combining *in vitro* cytokine assays with *ex vivo* drug penetration studies and *in vivo* functional evaluations has accelerated the development of targeted therapies for vesicant-induced injuries. Advanced techniques, such as RNA sequencing and proteomics, and imaging modalities, such as confocal microscopy and optical coherence tomography, further enrich these models (Kumar et al., 2024a, 2024b; Mohan et al., 2024; Sinha et al., 2025b). These tools provide comprehensive insights into the pathophysiology and therapeutic mechanisms of vesicant-induced ocular injuries, paving the way for innovative and effective treatments.

#### 4.1.1. In vitro corneal injury models

*In vitro* models rely on primary corneal cells or immortalized cell lines derived from the corneal epithelium, stroma, or endothelium (Kumar et al., 2023b). These models are commonly used to investigate the molecular mechanisms of vesicant toxicity, cellular stress responses, and drug screening. Specific assays can measure oxidative stress, inflammatory mediator release, apoptosis, and cytotoxicity, making these systems highly versatile for mechanistic studies (Kaluzhny et al., 2020; Kumar et al., 2023a; Mishra et al., 2023; Zhylkibayev et al., 2023).

The key advantages of ocular *in vitro* models include a high level of experimental control, which enables the precise manipulation of variables. They are cost-effective, reproducible, and particularly suitable for high-throughput drug screening. Moreover, they eliminate ethical concerns associated with animal experimentation (Chalmers et al., 2023). However, their major limitations include a lack of structural and functional complexity, as they cannot replicate the multilayered architecture of the cornea or its interactions with systemic responses. Despite these limitations, *in vitro* models have proven invaluable for preliminary investigations (McKay et al., 2019). *In vitro* models have enabled detailed studies of senescence (Anwar et al., 2024), SMAD2/3 signaling (Sinha et al., 2023), PI3K signaling (Sinha et al., 2025b), in situ structural and cellular aberrations (Sinha et al., 2022), and DNA damage and inflammation pathways (Goswami et al., 2016b; Zhylkibayev et al., 2023) in corneal cell lines following SM, NM and PAO injury. Thus, the cell viability study of human corneal epithelial cells exposed to PAO demonstrated cell death through ferroptosis and reduced proliferation, closely aligning with the severe damage observed in corneal epithelial cells in the rabbit *ex vivo* cornea model and accurately mimicking the expression of damage-associated molecular markers (Kandhari et al., 2023).

*In vitro* corneal models provide a controlled platform to dissect SM-induced molecular mechanisms and rapid screening of candidate MCMs before advancing to complex *ex vivo* or *in vivo* systems. The ability to correlate molecular signatures such as ferroptosis induction, PI3K/SMAD pathway activation, or DNA damage responses, between *in vitro* systems to physiologically relevant *in vivo* models enhance their bench-to-bedside translational. Future refinements, including three-dimensional cultures or organ-on-chip platforms mimicking cornea’s multilayered architecture, tear film dynamics, and immune-neural interactions, could bridge the gap between mechanistic discovery and clinical interventions.

#### 4.1.2. Ex vivo corneal injury models

*Ex vivo* models use organ-cultured corneas obtained from human donors or animals, such as rabbits, mice, and equines. These models preserve the anatomical integrity of the cornea, including its layered architecture and cellular heterogeneity, making them highly suitable for short-term studies of vesicant-induced toxicity, drug penetration, and stromal-epithelial interactions. Advantages of *ex vivo* models include their ability to maintain corneal architecture, enabling realistic modeling of injuries and therapeutic effects. They are also highly useful for evaluating drug permeability and tissue-level responses to vesicants. However, the viability of corneal tissues is restricted to few days, which limits their use in long-term studies. In addition, they lack systemic immune and vascular interactions, which are critical for modeling chronic injuries.

*Ex vivo* rabbit ocular injury models have been used extensively to assess the effects of NM exposure on corneal tissue integrity and biochemical alterations. A previous study investigated the impact of NM exposure (100 nmol for 2 h) in culture medium, with evaluations conducted 24 h post-exposure (Tewari-Singh et al., 2012). The findings revealed significant structural and molecular disruptions, including increased corneal epithelial thickness, corneal ulceration, microbullae formation, and separation between the epithelium and stromal layers. Additionally, apoptotic cell death was prominent, and key inflammatory and angiogenic markers, such as vascular endothelial growth factor (VEGF), COX-2, and MMP-9, were upregulated in the corneal epithelium, indicating a severe inflammatory response. The same groups subsequently examined NM-induced corneal damage at various concentrations (50–200 nmol in 10 mL of media), with a longer evaluation period of 48 h post-exposure (Goswami et al., 2016b). Like previous studies, corneal epithelial thickening and epithelial–stromal detachment were observed, along with an increase in apoptotic activity. This study further corroborated the role of VEGF, COX-2, and MMP-9 in exacerbating inflammatory and structural damage, highlighting the progressive nature of NM-induced ocular injury over time. The findings emphasize the importance of extended post-exposure evaluations to capture the full extent of corneal damage and inflammatory progression.

In an organ culture model, whole intact rabbit eyes were exposed to NM at different concentrations (0, 1, 2.5, 5, or 10 mg/mL) via topical application of a 5 mm filter disc for a variety of durations (5, 10, or 15 min), with a study endpoint of 48 h post-exposure (Charkoftaki et al., 2018). This study provides insights into NM-induced biochemical alterations, demonstrating extensive corneal epithelial degradation alongside significant increases in sphingomyelins, ceramides, and diacylglycerols. These findings suggest that lipid metabolism dysregulation plays a crucial role in NM-induced ocular toxicity, potentially contributing to barrier dysfunction and corneal damage. Collectively, the *ex vivo* models provide critical mechanistic insights into NM-induced ocular injury and serve as valuable platforms for evaluating therapeutic interventions.

In other study of VS-induced corneal damage, the authors revealed that the treatment of *ex vivo* rabbit corneas with arsenical vesicant PAO (5 or 10 μg) for 3, 5, and 10 min causes moderate to extensive corneal epithelial layer degradation and reduced the epithelial layer thickness in a concentration- and time-dependent manner (Kandhari et al., 2023). This study has also demonstrated a similarity in PAO-induced injuries of human corneal epithelial cells and cultured rabbit cornea.

**Fig. 5.**
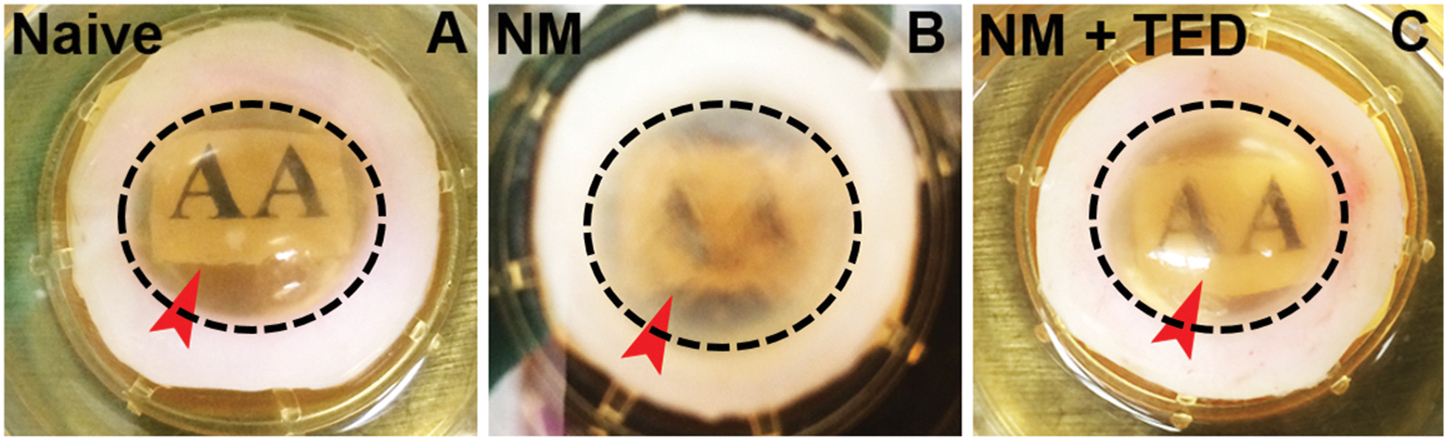
*Ex vivo* human corneal organ culture demonstrates the protective effects of TED against NM-induced toxicity. (A) Naïve corneas retained full transparency, allowing clear visualization of the underlying text. (B) NM-exposed corneas exhibited severe opacification, obscuring the text and indicating significant corneal damage. (C) TED-treated corneas showed reduced opacity and improved transparency, preserving refractive properties and enabling text recognition.

Using an *ex vivo* human corneal organ culture model, the therapeutic efficacy of topical eye drops (TED) consisting of four FDA-approved drugs, Ketorolac, Enalapril, Suberoylanilide hydroxamic acid (SAHA or vorinostat), and Vitamin C, against NM toxicity was assessed by Mohan’s group (Tripathi et al., 2020). In this model, NM (200 μM) was topically applied onto the central donor human cornea for 30 s via a 6-mm filter disc, followed by saline washing and incubation of tissue in an humidified CO_2_ incubator for 2 h. TED was then administered topically every 8 h for 3 days while maintaining the corneas under *ex vivo* culture conditions. Corneal transparency was evaluated by placing a text sheet beneath the cornea and analyzing the visibility (Fig. 5). Naïve corneas (Fig. 5A) remained transparent, allowing clear text visualization, while NM-exposed corneas (Fig. 5B) exhibited severe opacity, obscuring the text. However, the TED-treated corneas (Fig. 5C) displayed significantly reduced opacity, preserving corneal transparency and refractive properties, enabling clear text recognition. A similar *ex vivo* model was implemented by Mohan’s group previously for *ex vivo* equine cornea to study corneal ECM integrity and cellularity over a 7-day culture period (Marlo et al., 2017). Two culture systems utilized an immersion condition (IC) system and an air–liquid interface (ALC) system. The ALC system was found to be superior in preserving corneal transparency and maintaining structural integrity with minimal stromal disorganization and apoptosis compared to the IC system, which resulted in severe corneal edema, epithelial loss, and significant α-SMA upregulation indicative of fibroblast activation.

These findings highlight the importance of optimizing *ex vivo* corneal models to maintain corneal homeostasis and provide valuable insights into corneal wound healing and fibrosis. Such models are highly relevant for studying human corneal injuries, including vesicant-induced damage, by offering a physiologically relevant platform for therapeutic testing and mechanistic evaluations.

*Ex vivo* corneal models provide an essential intermediate platform between cell-based systems and *in vivo* studies and offer realistic structural context for assessing vesicant-induced ocular pathology and therapeutic responses. Their ability to preserve corneal architecture allows detailed evaluation of barrier disruption, stromal-epithelial crosstalk, and extracellular matrix remodeling under controlled conditions. Importantly, correlations between *ex vivo* findings and in animal models reinforce their translational relevance.

#### 4.1.3. In vivo corneal injury models

*In vivo* models are considered most suitable for studying vesicant-induced ocular injuries due to precisely controlled experimental conditions. *In vivo* models allow for the study of the time-dependent progression of injuries, encompassing both the acute, delayed, and chronic phases. Additionally, these models simulate systemic interactions, such as immune cell infiltration and vascular responses, which are critical for understanding the full spectrum of vesicant’s toxicological effects to eyes. Furthermore, *in vivo* models provide unparalleled insights into the efficacy of systemic and topical therapies. Rabbit eyes are preferred model for corneal studies because of their physiological and anatomical similarities to the human cornea while mice have been widely used to study the vesicant’s toxicity to the cornea because of the low-cost, easy-handling, and availability of a wide range of transgenic knock-in and knock-out lines.

##### 4.1.3.1. Exposure of SM to cornea.

Rabbit eyes and human eyes exhibit similar lesions at functionally equivalent doses of SM despite being 4–6 times less sensitive to SM toxicity compared to the human eyes (Gates and Moore, 1946; Goswami et al., 2021; Mann and Pullinger, 1942; McNutt, 2023; Papirmeister et al., 1991b; Sinha et al., 2022). However, the advantages of *in vivo* models must be balanced against their relatively high costs, ethical concerns, and interspecies differences in immune responses and healing rates which may reduce translational relevance.

In an early study by Livingston and Walker, liquid SM (0.75 μL) was directly applied to the rabbit cornea for 1 min, with observations recorded over a 28-day period (Livingston and Walker, 1940). The exposure led to severe corneal epithelium disruption, ulcer formation, and damage extending through all the corneal layers, including the endothelium. Inflammatory responses were evident, with symptoms such as photophobia, mucous discharge, conjunctival blistering, and iris contraction. Mann and Pullinger conducted similar exposures with small aliquots of SM distributed across 3–9 locations of the cornea and monitored over a year (Mann and Pullinger, 1942). They also report full-thickness corneal lesions with a biphasic injury progression. Furthermore, they then correlated pathophysiologies observed in rabbits to clinical cases of SM poisoning in war veterans. Collectively, these reports are the earliest demonstration of an animal model that reproduces many of the acute and long-term clinical effects of SM exposure on ocular integrity and function.

Petrali et al. investigated the acute effects of liquid SM (0.4 μL) on corneal ultrastructure at 24 h post-exposure (Petrali et al., 1997). The results showed significant epithelial and stromal damage, including edema, necrosis, and epithelial degradation. Cellular-level alterations were also noted, with pyknosis and degenerative acantholysis contributing to the breakdown of the corneal structure. In a subsequent study, a larger volume of liquid SM (400 μL) was applied to the cornea, with similar observations recorded at the 24-h post-exposure mark (Petrali et al., 2000). Although these studies used a super-physiological SM dose and should be interpreted with caution, the authors found nuclear pyknosis, epithelial cell death, and loss of polarity as key indicators of cellular distress. Additional findings included epithelial–stromal separation, stromal edema, and fibroblast degeneration, all of which contribute to a compromised corneal structure. Additionally, disruptions in protein expression patterns (laminin, Ki67, p53, and desmosomal proteins) were detected, which suggests that SM exposure alters the molecular signaling pathways involved in corneal repair and integrity.

Acknowledging that large-scale casualties would likely necessitate vapor exposure, Kadar et al. developed a goggle-based vapor exposure method and explored the long-term effects of SM vapor on the eye at two doses (740 mg min/m^3^ and 840 mg min/m^3^), with evaluations extending up to three months post-exposure (Kadar et al., 2001). Acute effects including ocular swelling, conjunctival hyperemia, and blepharospasm. Corneal inflammation led to oxidative stress, corneal epithelial degradation, and infiltration of inflammatory cells into the stromal layer. After partial resolution, a dose-dependent delayed onset of corneal pathologies was also observed, reproducing the biphasic injury observed clinically. The same group next modified their exposure system to deliver SM vapor from SM liquid-impregnated filter paper mounted inside protective goggles (Kadar et al., 2009). Because the authors did not measure the vapor concentration over time, the applied dose was uncertain. In clinical assessments conducted for up to three months post-exposure, this exposure method elicited a delayed injury in ~50 % of eyes. The group found an increase in inflammatory markers in the cornea, such as prostaglandin E2 and calcitonin gene-related peptide (CGRP), in eyes that developed a delayed injury compared to eyes that resolved. Using the same exposure method, this group found that SM vapor elicited corneal nerve degeneration with a Wallerian-like pattern, along with alterations in calcitonin gene-related peptide (CGRP) levels and the onset of limbal stem cell deficiency (LSCD) (Kadar et al., 2013). Interestingly, limbal stem cell dysfunction manifested weeks after mustard exposure, suggesting that limbal injury may occur secondary to mustard toxicity, such as through limbal exhaustion or immunotoxicity in the limbal niche (Kadar et al., 2011, 2022). The loss of corneal nerve function and disruption of the limbal stem cell niche indicated that SM exposure not only affects the corneal structure but also impairs its regenerative capacity, potentially leading to persistent ocular dysfunction. This group also reported the activation of Th1 and Th2 immune pathways at 1 month after exposure, indicating a complex inflammatory response (Horwitz et al., 2019). Notably, the IL-6 and ERK5 signaling pathways were upregulated exclusively in corneas that exhibited clinical symptoms, associating sustained pro-inflammatory signaling with MGK.

Milhorn et al. further refined the vapor exposure model by developing a cornea-specific vapor cap exposure method (Milhorn et al., 2010). Because MGK symptoms primarily manifest in the cornea, the authors argued that reducing extra-ocular injuries (and associated pain and distress) would allow improved understanding of corneal injury processes. They first characterized the long-term effects of a 2.5 min SM vapor exposure on rabbit eyes (approximately 1500 mg min/m^3^), with observations extending up to 16 weeks post-exposure. This study revealed a persistent inflammatory response characterized by an increase in key cytokines, including IL-1β, TNF-α, IL-6, and IL-8. Matrix metalloproteinases MMP-2 and MMP-9 levels were sustained over months, suggesting ongoing tissue remodeling and degradation within the cornea. McNutt et al. followed this foundational study by investigating the histological, microscopic and biochemical effects of a 2.5 min SM vapor exposure on the cornea over an eight-week period (McNutt et al., 2012). The study confirmed increased protein levels of MMP-2, MMP-9, IL-1β, TNF-α, IL-6, and IL-8, which are associated with significant structural damage. Observations included destabilization of the basal corneal epithelium, basement membrane irregularities, and stromal deformation accompanied by edema and recurrent corneal erosions. The presence of basal cell necrosis further underscored the cytotoxic effects of SM, emphasizing its potential to disrupt corneal integrity over time. In a subsequent study, the same group focused on the endothelial layer of the cornea and its vulnerability to SM exposure (McNutt et al., 2013). The study, which followed subjects for eight weeks after exposure, was the first to establish a link between endothelial toxicity and MGK. These findings suggest that endothelial injury plays a crucial role in the pathophysiology of SM-induced MGK, particularly after high-dose SM exposure, whereby endothelial barrier failure directly contributes to long-term vision impairment (McNutt et al., 2016). McNutt et al. also examined the long-term impact of SM vapor exposure over a range of corneal volumes using different vapor cap sizes (9, 11, and 14 mm) (McNutt et al., 2020b). The study found a strong correlation between the exposed corneal volume and the likelihood of MGK emergence. Based on further correlations between endothelial barrier integrity and cap size, the authors hypothesized that the extent of corneal endothelial cell loss was one factor that determined whether SM-exposed corneas resolved or developed MGK.

In an elaborate study of SM vapor dose responses on the cornea, McNutt et al. described acute and chronic responses after corneal exposure to SM at saturating vapor pressures for various durations (30–150 s, producing estimate vapor doses of 300–1500 mg min/m^3^) (McNutt et al., 2021). These data showed acute signs of corneal damage emerge after a 30 s exposure and reach a plateau between 90 and 120 s. Furthermore, the group found that doses of 60 s or higher produced a delayed lesion that emerged 4–8 weeks after the acute injury (e.g., following a subacute phase) and lasted for approximately three weeks in duration. This recurrent edematous lesion (REL) was characterized by severe corneal edema and epithelial erosions, which were followed by irreversible pathophysiological responses such as corneal scarring and neovascularization. Notably, whereas the frequency of REL development was dose-dependent, the severity of the REL was not dose-dependent. In a related review article (McNutt, 2023), McNutt hypothesized the REL is a maladaptive response to the acute lesion and, furthermore, that medical countermeasures that prevent REL would attenuate or prevent MGK symptoms. Although the development of effective treatments may require a greater understanding of this complex pathophysiological progression, multiple therapeutic interventions during the subacute period have shown therapeutic benefit, consistent with this hypothesis (Gore et al., 2018, 2021; Horwitz et al., 2018; Panahi et al., 2017, 2018; Shalwitz et al., 2023; Tripathi et al., 2020).

**Fig. 6.**
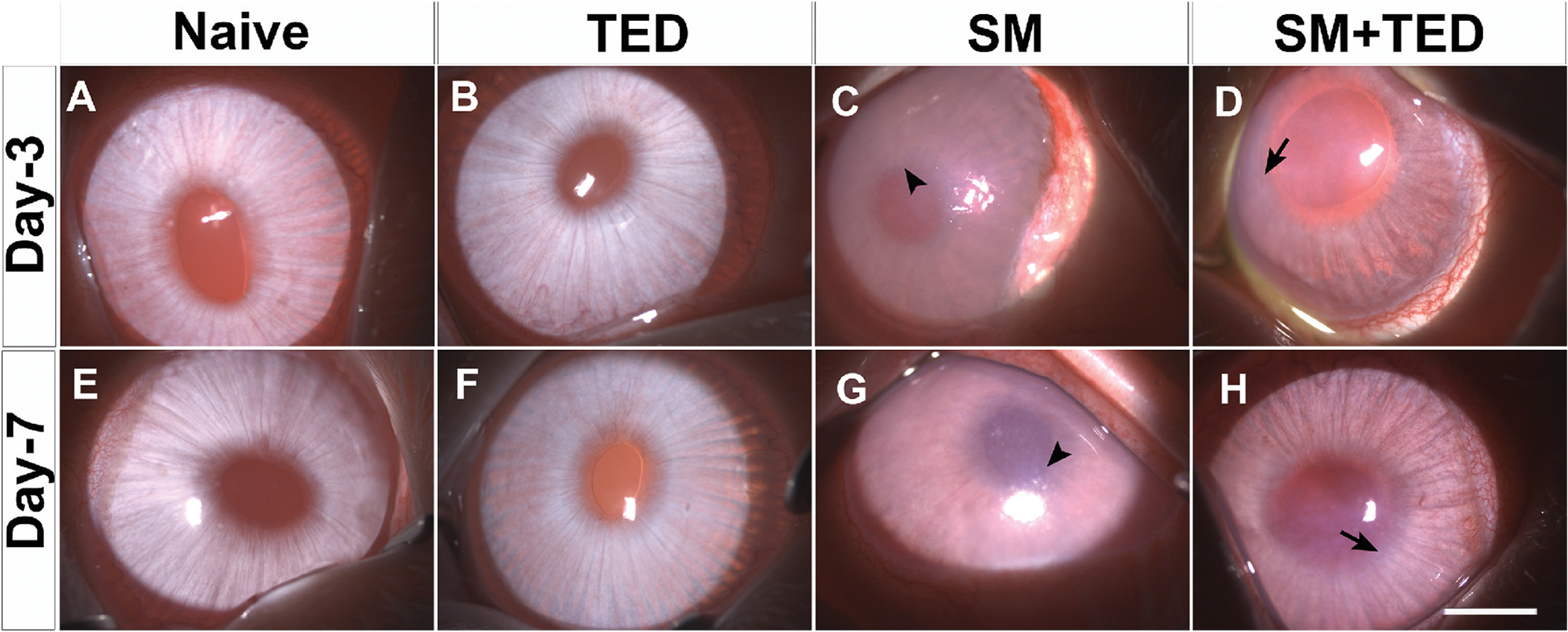
Representative stereomicroscopy images illustrating corneal opacity. SM vapor exposure induced severe corneal haze in rabbits at day 3 (C, arrowhead) and day 7 (G, arrowhead) *in vivo*. TED treatment effectively reduced SM-induced haze at day 3 (D, arrow) and day 7 (H, arrow). TED application on SM-unexposed eyes caused no corneal haze or inflammation on day 3 (B) or day 7 (F), maintaining ocular health comparable to naïve corneas at day 3 (A) and day 7 (E). Scale bar: 2.0 mm (adapted from Tripathi et al., 2020).

A recent *in vivo* study by Tripathi et al. evaluated the therapeutic potential of a novel multimodal ophthalmic formulation, TED, in mitigating acute MGK in a rabbit model (Tripathi et al., 2020). Using a rabbit model, the study exposed eyes to SM vapor at an estimated dose of 1600 mg min/m^3^ and monitored structural and molecular changes at 3- and 7-days post-exposure. Rabbits treated with TED, a combination of ketorolac, SAHA, enalapril, and vitamin C, exhibited reduced corneal inflammation, epithelial–stromal separation, and edema compared to untreated SM-exposed eyes (Fig. 6). TED also downregulated inflammatory markers, such as COX-2 and TGF-β1, which are associated with corneal fibrosis and prolonged injury. These results indicate that TED could help preserve corneal integrity and mitigate the early toxic effects of SM exposure.

Joseph et al. investigated the effects of liquid SM exposure (0.4 μL; 5 mg ≈ 30 nmol) on corneal and extraocular tissues, with observations extending to 28 days post-exposure (Joseph et al., 2021). The study revealed increased keratin 17 expression in corneal epithelial cells located above the basement membrane, suggesting an abnormal wound-healing response. Additionally, there was an increase in neutral mucin-producing goblet cells and elevated expression of the membrane-associated mucins MUC1 and MUC4 in the conjunctival epithelium.

Several studies also explored ocular morbidities over longer time intervals using mouse and rabbit models. A mouse model was used to study a 30 s corneal vapor exposure on ocular health, with observations extending up to one year post-exposure (Ruff et al., 2013). This study revealed biphasic progression of corneal injury. The acute phase is characterized by damage to the corneal epithelial and limbal regions, accompanied by redness, swelling, and inflammation. Following an intermittent recovery phase, a latent period ensues, eventually leading to the delayed onset of MGK (Tripathi et al., 2020; Sinha et al., 2025b). The symptoms observed in mice closely mirrored those documented in rabbits, which suggests a conserved pathological response across species. A recently completed up to 12-month study in SM-exposed rabbits further revealed both chronic and delayed toxic effects, providing critical insights into the long-term progression of SM-induced ocular damage (Mohan et al., unpublished). The findings indicated that while acute exposure triggers immediate corneal epithelial erosion, edema, and inflammation, the long-term effects manifest as progressive corneal thinning, stromal fibrosis, neovascularization, and persistent epithelial defects that resemble the mustard MGK observed in human cases. The similarities between rabbit and mouse *in vivo* models highlight the conserved pathological response across species, arguing both species may contribute to understanding SM-induced ocular toxicity and its long-term consequences.

*In vivo* SM corneal injury animal models have advanced our understanding of both acute and delayed manifestations of MGK and a biphasic nature mirrors human pathology. Early rabbit and mouse studies established the reproducibility of key clinical hallmarks, epithelial disruption, stromal edema, ulceration, and delayed-onset neovascularization while later refinements, such as vapor cap systems and cornea-specific exposures, minimized extraocular involvement and allowed for more precise dissection of corneal pathophysiology. Across models, a consistent pattern emerges in which acute epithelial and stromal damage is followed, after a latent phase, by recurrent edematous lesions, stromal remodeling, and endothelial compromise. The translational relevance of these findings is strengthened by the identification of shared molecular drivers such as sustained MMP activity, persistent pro-inflammatory cytokine production, and limbal stem cell dysfunction, which may offer potential therapeutic targets. Notably, interventional studies in the subacute phase, such as TED therapy, support the hypothesis that timely modulation of inflammation and fibrosis could prevent chronic MGK progression. Also, these *in vivo* models are valuable for studying dose-response relationships, validating biomarkers, prediction of delayed lesions, and finding MCMs with prophylactic and therapeutic potential.

##### 4.1.3.2. Exposure to NM.

Ocular exposure to NM also results in both acute and delayed phases of corneal disruption. The effect of aqueous NM solution on the blood-aqueous humor barrier was first reported by Davson and Quilliam in 1947 in rabbit eyes (Davson, 1947). Subsequent studies by other researchers further showed NM-induced miosis and weakening of the aqueous humor barrier (Crawford and Matrisian, 1996; Jampol et al., 1976). In addition to these manifestations, these investigations also described ocular irritation, ischemia, and ocular hypertension as biphasic injuries in treated rabbit corneas. More recently, Goswami et al. employed a topical NM application protocol—100 μL of a 1 % solution applied for 5 min—and evaluated the effects in rabbit eyes over a 28-day period (Goswami et al., 2019). This study also reported edema of the conjunctiva and eyelids, corneal opacity and ulceration, corneal thickening, epithelial degradation, increased in blood vessel and inflammatory cell counts, decreased keratocyte numbers in corneal stroma. Moreover, the study found a similarity in molecular signaling induced by NM between *in vivo* and *ex vivo* rabbit corneal models (Goswami et al., 2016b). In a separate murine model of delayed-onset mustard keratopathy, *in vivo* exposure to NM was shown to induce cellular senescence in corneal tissues. The degree of senescence positively correlated with fibrosis severity, implicating senescence-associated pathways as possible targets for mitigating chronic corneal remodeling (Soleimani et al., 2023).

To investigate the acute response of mouse corneal tissue to NM exposure, the Gorbatyuk group conducted a study in which mice were treated with 80 μg of NM applied to the corneal surface for 3 min using an eye patch (Zhylkibayev et al., 2024). Histological analysis during the acute phase revealed a significant increase in corneal thickness in the treated eyes, indicative of corneal edema. This was accompanied by a marked increase in TUNEL-positive cells, providing clear evidence of corneal cell death.

In a separate study, Nalbant et al. applied a 0.5 % NM solution to the mouse eye using 2 mm filter paper discs for 1 min (Kayaalp Nalbant et al., 2025). They reported corneal haze in the treated eyes, with an average score of 1.9 at one-week post-exposure, reflecting acute phase injury. Fluorescein dye staining and cobalt blue light visualization revealed disrupted corneal epithelium characterized by thinning, disorganization, and focal areas of complete epithelial loss by day 3 post-exposure. By four weeks post-exposure (the delayed phase), most areas of the cornea were reported to be vascularized.

The corneal ulceration and neovascularization, occurring in a dose dependent manner of NM exposure has been also reported in Sprague Dawley rats (Poudel et al., 2024).

##### 4.1.3.3. Exposure to arsenical vesicants to cornea.

Lewisite-induced corneal injury has been studied in rabbits by the research group led by Agarwal et al. by exposing animals to lewisite vapor at a concentration of 0.2 mg/L (Tewari-Singh et al., 2016). The rabbits were exposed for varying durations: 2.5, 5.0, 7.5, and 10.0 min. Clinical progression of the injury was monitored for up to 28 days in a dose-response study and for up to 56 days in a time-response study. The authors observed that increasing durations of lewisite exposure resulted in corneal opacity developing within 6 h post-exposure. This opacity intensified over the first 3 days, then gradually declined over the next three weeks, before increasing again — suggesting a multiphasic progression of corneal injury. Lewisite -induced corneal ulceration peaked at 1 day post-exposure and exhibited a recurring pattern thereafter. In addition, neovascularization of the cornea began at 7 days post-exposure, peaked between days 22 and 35, and persisted beyond this period. Other clinical manifestations included increased corneal thickness, iris redness, swelling and redness of the conjunctiva (Tewari-Singh et al., 2016). Overall, this study is the first to establish clinically relevant and quantitative endpoints for lewisite-induced ocular injury in rabbits.

In a more detailed follow-up analysis, the same research group further investigated the histopathological features of lewisite-induced corneal damage (Tewari-Singh et al., 2017a). Histological examination of the affected corneas revealed a significant increase in the number of blood vessels (neovascularization) and infiltration of inflammatory cells, accompanied by a concurrent decline in keratocyte density. These effects were most pronounced at day 7 post-exposure, particularly following the 7.5-min lewisite exposure. Additionally, a marked epithelial-stromal separation was observed on days 7 and 14 post-exposure, indicating structural disruption of the corneal architecture. These histopathological changes corroborated the clinical findings and underscored a dynamic and progressive injury pattern following lewisite exposure. Overall, this study established key histological and clinical endpoints of lewisite-induced corneal injury across both lower and higher exposure durations. The rabbit model used in this study provides a valuable platform for future research focused on the development and evaluation of therapeutic interventions targeting lewisite-induced ocular damage (Tewari-Singh et al., 2017a).

The Gorbatyuk group recently investigated the effects of PAO, a surrogate for lewisite, on the mouse eye using an eye patch exposure method (Zhylkibayev et al., 2023). In this study, the researchers found that PAO exposure activated molecular pathways associated with corneal cell death, mirroring those triggered by exposure to mustard agents and lewisite. Importantly, they observed a significant increase in corneal tissue thickness at 72 h post-exposure, a hallmark of chemically induced ocular injury. These findings highlight the pathophysiological relevance of PAO-induced corneal damage and support the use of this model for further investigation into the cellular signaling pathways involved. Moreover, this study underscores the potential utility of PAO as a surrogate agent in the development and screening of therapeutic countermeasures to mitigate the ocular consequences of VS exposure (Zhylkibayev et al., 2023).

### 4.2. Models of retinal injury

Previous studies from Gorbatyuk’s group, along with findings from other investigators, have shown that direct ocular exposure (DOE) to vesicants causes severe, dose-dependent retinal injury in mice (Basu et al., 2024; Zhylkibayev et al., 2024, 2025). This damage arises from disruptions in cellular signaling, ultimately leading to retinal degeneration. In cases of systemic exposure in humans, retinal injury can occur through two primary pathways: 1) direct transmission and/or penetration of the traveling molecular signal and/or the vesicant itself via corneal exposure, and 2) indirect exposure through ocular blood circulation (Fig. 1). Recent study including our published work have demonstrated that both direct ocular and skin exposure to vesicants alter the plasma metabolic profile and induce ocular tissue inflammation in mice (Bouhlel et al., 2024; Zhylkibayev et al., 2023). These findings suggest that signaling molecules generated in response to corneal exposure to vesicants may be secreted, travel through local or systemic pathways, and subsequently affect the neighbor ocular tissue and cells.

The alkylating nature of mustard agents induces DNA damage, leading to retinal cell death. Arsenical vesicants, such as PAO and lewisite, react with thiol groups in proteins, forming stable ring structures that inhibit the function of many thiol-containing enzymes (Li et al., 2016a). The primary pathophysiological effects of vesicant-induced retinal damage are attributed to direct cytotoxicity to neuronal cells driven by oxidative stress and inflammation. For example, retinal ganglion cells (RGCs) are particularly vulnerable to DOE due to their proximity to blood flow along the path of warfare agents. Similarly, the retinal pigment epithelium (RPE) and photoreceptor (PR) cells are highly sensitive to exposure because of their high metabolic activity and dependence on blood vessels, which can carry excessive reactive oxygen species. Additionally, a compromised retinal microvascular system may lead to neovascularization and blood leakage. In such cases, damaged blood vessels exacerbate retinal inflammation by attracting proinflammatory cytokines and immune cells. Over time, this process results in retinal scarring and edema. Collectively, these events can disrupt photoreceptor function and impair RPE integrity. The *in vitro* and *in vivo* models used to study the retinal injury are summarized in Table 1.

#### 4.2.1. In vitro retinal injury model

Several experimental retinal models can be developed to study vesicant-induced retinal damage. Among them are *in vitro* models, which include commercially available human retinal endothelial cells (HREC) and human retinal pigment epithelial cells (ARPE-19), as well as cell lines accessible from investigators, such as cone-derived 661W and Müller glial (MIO-M1) cells. The advantages of using these cellular models in vesicant toxicity studies lie in their accessibility, reproducibility, and reliability. Additionally, *in vitro* assays are relatively inexpensive and provide valuable insights into the molecular mechanisms of cell toxicity. Furthermore, the broad range of toxicological testing options enables better control over experimental conditions, precise validation of concentrations, and analyses across multiple time points with various doses. For instance, MIO-M1 cells were used in a recent study to investigate the role of mustard toxicity (Mahaling et al., 2023). In this study, MIO-M1 cells were treated with different concentrations (50–500 μM) of NM (HN3, Tris (2-chloroethyl) amine), an experimental analog of SM, for 3, 24, and 72 h. The authors identified the activation of caspase-1 and the NLRP3 inflammasome, leading to pyroptotic cell death. Recently, the Gorbatyuk’s group utilized primary HREC cells treated with 100 μM of NM (HN2, Bis (2-chloroethyl) amine) and observed activation of the UPR PERK pathway (Zhylkibayev et al., 2024). Key PERK arm proteins, such as GADD34 and phosphorylated eIF2α, were significantly upregulated 24 h post-exposure (Zhylkibayev et al., 2024). Sustained activation of the UPR PERK pathway is a hallmark of cellular stress and can lead to apoptotic cell death. These findings were consistent with the previous studies from this team employing phenylarsine oxide (PAO), a lewisite surrogate, to elucidate the molecular mechanisms of vesicant exposure in treated corneal cells (Zhylkibayev et al., 2023) and skin keratinocytes (Srivastava et al., 2016).

*In vitro* models allow researchers to dissect the mechanisms of vesicant-induced apoptotic retinal cell death, which involves the activation of inflammatory signaling pathways and a sustained UPR. Expanded research should be conducted to verify the dose-dependent responses of distinct cultured retinal cells—such as cone-derived 661W cells, Müller glia, and retinal pigment epithelial cells—to various vesicants, including SM, NM, and lewisite. These studies will help elucidate cell-specific vulnerabilities and mechanisms of toxicity, ultimately guiding the development of targeted therapeutic strategies.

#### 4.2.2. In vivo retinal injury models

*In vivo* mouse models have been extensively used to study the effects of vesicant exposure (Basu et al., 2024; Joseph et al., 2016; Li et al., 2016c; Manzoor et al., 2020; Srivastava et al., 2024; Tewari-Singh and Agarwal, 2016). In particular, vesicant-induced retinal injury has been explored by Gorbatyuk’s team, who studied the effects of lewisite, PAO, and NM (Zhylkibayev et al., 2023, 2024), as well as Mandal et al., who investigated the DOE of NM in mice (Basu et al., 2024). Mouse models offer several advantages over other animal models, including cost effectiveness, reproducibility, and reliability. Additionally, the availability of genetically modified mice further enhances their utility for studying disease mechanisms and therapeutic interventions. For example, the sustained upregulation of UPR could be further investigated using PERK, ATF4, and TRIB3-deficient mice in studies involving DOE exposure to vesicants. Another example includes examining the role of VEGF signaling in injured corneal tissue, which could be assessed using mice with modulated VEGF expression or secretion; such mice are available from commercial sources. Despite these advantages, the use of mouse models over larger animal models (such as pigs or rabbits) has several drawbacks. These include anatomical and physiological differences from the human eye, smaller ocular structures that may limit certain assessments, and differences in metabolic and immune responses, which can impact the translation of findings to humans.

## 5. Pathobiology of corneal injury following exposure to vesicants

### 5.1. Effects of vesicants on corneal and vascular tissue

Animal models have significantly contributed to our understanding of the ocular pathology resulting from vesicant exposures, offering translational insights into clinical observations.

*In vivo* rabbit studies have demonstrated that vesicant exposure causes dose-dependent corneal damage, observable during clinical evaluations as epithelial sloughing, stromal edema, vascular congestion, stromal haze, and neovascularization (Kadar et al., 2001; Kadar et al., 2009; McNutt et al., 2012a; McNutt et al., 2013; McNutt et al., 2021; Tripathi et al., 2020). Vesicants such as mustards, lewisite, and phosgene oxime are lipophilic and readily penetrate the cornea, often inducing cytotoxicity in epithelial, neuronal, keratocyte, and endothelial cell populations without immediately disrupting gross corneal architecture (Axelrod and Hamilton, 1947; Kadar et al., 2009; McNutt et al., 2013; McNutt et al., 2020b). Clinical imaging tools like OCT and slit-lamp evaluations reveal acute corneal clouding, persistent epithelial defects, and chronic stromal thinning during later stages, indicative of biphasic injury progression.

Microscopic analysis of rabbit corneas post-vesicant exposure has revealed histopathological changes including keratocyte apoptosis, loss of epithelial integrity, and endothelial dysfunction. Within one week after exposure, epithelial regeneration typically occurs, forming an intact though immature barrier, accompanied by reductions in corneal edema and opacity (McNutt et al., 2012a; McNutt et al., 2012b). However, some corneas transition into a subacute phase and later develop delayed-onset pathology characterized by stromal fibrosis, neovascularization, and persistent epithelial breakdown hallmarks of MGK (McNutt et al., 2021).

Following vesicant exposure, disruption of the epithelial–stromal barrier permits infiltration of inflammatory cells such as neutrophils and macrophages from the tear film. These cells release a range of proinflammatory cytokines and chemokines including IL-1α, IL-1β, IL-6, IL-8, IL-20, IL-33, MCP-1, and VEGF (Goswami et al., 2019, 2021; Horwitz et al., 2019; Kadar et al., 2014). These molecular events amplify tissue damage and initiate cascades of oxidative stress and immune activation. Over time, stromal remodeling is marked by collagen disorganization, excessive extracellular matrix deposition, and myofibroblast activation, all of which contribute to corneal scarring and reduced visual acuity (Kumar et al., 2023b; Mohan et al., 2022).

Endothelial dysfunction is another critical pathology associated with vesicant exposure. Early studies using liquid vesicants in rabbit eyes reported corneal endothelial abnormalities, including desquamation from Descemet’s membrane (Livingston and Walker, 1940; Mann and Pullinger, 1942). More recent *in vivo* rabbit studies have confirmed that high-dose sulfur mustard vapor can penetrate the cornea and affect deeper ocular structures. Using radiolabeled SM, Kadar et al. demonstrated accumulation in the lens and ciliary body, highlighting SM’s ability to traverse the corneal barrier (Kadar et al., 2009).

Functional assessments combined with multimodal imaging techniques have shown that SM exposure induces acute endothelial toxicity, followed by chronic morphological changes in corneal endothelial cells (McNutt et al., 2013). These changes include loss of endothelial barrier function, retrocorneal fibrous membrane deposition, a hallmark of endothelial failure and immune cell infiltration of the posterior cornea (McNutt et al., 2020b, 2021). In some cases, persistent damage was associated with endothelial-to-mesenchymal transition (EndoMT), leading to endothelial cell death and failed regeneration. These pathological changes are believed to contribute to the emergence of MGK-like symptoms in rabbit models.

Similar findings have been observed in humans. Histological analyses of MGK patients revealed reduced corneal endothelial cell (CEC) density, alongside increased cell size variability and morphological abnormalities (Jafarinasab et al., 2010). Because adult human CECs do not divide *in vivo*, their loss is irreversible. Compensatory cell spreading can temporarily restore the barrier, but if the injury exceeds regenerative capacity, persistent corneal edema and anterior keratopathies result (Joyce et al., 1996; Senoo et al., 2000; Fuchs et al., 2021; Petroll et al., 1995).

In addition to endothelial injury, oxidative stress and inflammatory mediators contribute to corneal nerve degeneration, leading to reduced sensitivity and delayed epithelial repair. This can result in neurotrophic keratopathy, which further compromises wound healing. Moreover, proangiogenic signaling, particularly involving VEGF, promotes corneal neovascularization (CNV) (Chang et al., 2012). CNV disrupts the cornea’s natural immune privilege, increasing the risk of immune rejection in corneal transplants and contributing to chronic inflammation and stromal opacification.

Histopathological findings in corneal tissue post-exposure revealed epithelial disruption, stromal edema, endothelial cell loss, inflammatory infiltration, fibrosis, and neovascularization. The loss of epithelial integrity is often accompanied by thinning, hyperplasia, and increased inflammatory cell infiltration, leading to persistent epithelial defects. Stromal fibrosis, which is mediated by activated myofibroblasts, results in disorganized collagen deposition, reducing corneal transparency. Endothelial dropout leads to progressive corneal edema and opacification, impairing vision. Inflammatory responses, characterized by macrophage and neutrophil infiltration, further contribute to tissue damage and delayed healing (Fuchs et al., 2021; Mohan et al., 2022). Stromal remodeling, a key pathological feature, is characterized by keratocyte apoptosis, collagen disorganization, and excessive deposition of extracellular matrix components. Moreover, myofibroblast activation contributes to corneal scarring, reducing transparency and visual acuity (Kumar et al., 2023b; Mohan et al., 2022). Clinically, these histopathological alterations manifest as corneal opacity, photophobia, recurrent erosions, and reduced visual acuity. Exposure to environmental and pathological insults induces profound structural and functional changes in corneal and vascular tissues. These alterations are driven by a complex interplay of epithelial, stromal, and endothelial dysfunction; inflammation; fibrosis; and aberrant neovascularization. Histopathological findings confirm the presence of cellular loss, tissue remodeling, and inflammatory infiltration, which are correlated with clinical manifestations, such as corneal opacity and vascular occlusion. Understanding these pathophysiological mechanisms is critical for developing targeted therapeutic interventions aimed at mitigating inflammation, restoring endothelial function, and preventing long-term fibrotic changes. Future research should focus on novel anti-inflammatory and antifibrotic strategies to improve clinical outcomes in patients with corneal injuries.

Clinically, these histopathological alterations manifest as corneal opacity, photophobia, recurrent erosions, and reduced visual acuity. Exposure to environmental pathogens and toxic chemicals to eyes induces structural and functional changes in cornea and ocular surface tissues. These alterations are driven by a complex interplay of epithelial, stromal, and endothelial cellular dysfunction; inflammation; fibrosis; and aberrant neovascularization. Histopathological findings confirm the presence of cellular loss, tissue remodeling, and inflammatory infiltration, which are correlated with clinical manifestations, such as corneal opacity and vascular occlusion. Understanding these pathophysiological mechanisms is critical for developing targeted therapeutic interventions aimed at mitigating inflammation, restoring endothelial function, and preventing long-term fibrotic changes. Future research should focus on developing MCMs with anti-inflammatory and anti-fibrotic strategies to improve clinical outcomes in patients with corneal injuries.

### 5.2. Molecular pathogenesis driving corneal tissue damage

Corneal tissue damage caused by vesicant exposure is driven by a complex cascade of molecular events that impair transparency, structural integrity, and regenerative capacity. These include oxidative stress, inflammation, extracellular matrix (ECM) remodeling, and programmed cell death, all of which are regulated by interconnected signaling pathways (Mohan et al., 2022). Upon exposure, reactive oxygen species (ROS) are rapidly generated, leading to lipid peroxidation, protein denaturation, and DNA damage. ROS activate redox-sensitive transcription factors such as NF-κB and AP-1, which amplify the expression of inflammatory cytokines and pro-apoptotic genes (Nita and Grzybowski, 2016; Tseng et al., 2013). Inflammatory mediators such as MCP-1 and CXCL8 recruit immune cells to the cornea, further intensifying tissue damage (Hu et al., 2014; Spandau et al., 2003). Concurrently, epithelial and stromal cell death compromises tissue integrity, delays wound healing and contributes to the development of chronic injury.

Several major signaling pathways have been implicated in the molecular pathogenesis of vesicant-induced corneal damage. The NF-κB pathway drives transcription of proinflammatory cytokines and adhesion molecules such as ICAM-1, facilitating leukocyte infiltration (Koganti et al., 2022; Mohan et al., 1998). The mitogen-activated protein kinase (MAPK) pathway including ERK1/2, JNK, and p38—regulates inflammation, apoptosis, and ECM remodeling (Anwar et al., 2024; Chen et al., 2022; Sinha et al., 2025a, 2025b). The PI3K/Akt pathway, frequently dysregulated following vesicant exposure, contributes to fibrosis and neovascularization through upregulation of LOX and VEGF. Matrix metalloproteinases (MMPs), particularly MMP-2 and MMP-9, degrade stromal collagen and undermine structural stability, while TGFβ signaling promotes myofibroblast differentiation and excessive ECM deposition, exacerbating stromal fibrosis (Joseph et al., 2021). Together, these signaling cascades create a self-perpetuating cycle of inflammation, tissue degradation, and fibrosis, ultimately driving the chronic progression of corneal injury following vesicant exposure.

### 5.3. Cellular and molecular mechanisms underlying corneal injury

The cornea, which is composed of the epithelium, stroma, and endothelium, relies on a highly organized cellular architecture to maintain its transparency and barrier function. When exposed to physical, chemical, or biological insults, corneal cells undergo rapid stress responses that can lead to either regeneration or progressive damage, depending on the severity and duration of exposure (Raut et al., 2024). The corneal epithelium, which serves as the first line of defense, often exhibits cytoskeletal disorganization, cell junction disruption, and apoptosis, compromising barrier integrity. Disruption of tight junction proteins, such as ZO-1, occludin, and claudins, leads to increased permeability and vulnerability to infection, whereas basal cell loss and limbal stem cell exhaustion impair regenerative capacity, resulting in delayed wound healing (Chen et al., 2012; Sugrue and Zieske, 1997).

The stromal layer, which is primarily composed of collagen and keratocytes, provides mechanical strength and optical clarity. Corneal injury induces keratocyte apoptosis, leading to stromal thinning and impaired extracellular matrix integrity. This process is mediated by Fas-FasL signaling, which triggers caspase-dependent apoptosis. Additionally, myofibroblast differentiation, driven by TGF-β, results in excessive ECM deposition and corneal fibrosis, contributing to stromal haze and scarring. Moreover, endothelial cell loss impairs the function of Na+/K + ATPase pumps, disrupting hydration control and resulting in corneal edema and visual impairment (Mohan et al., 2024).

Recently, Kumar et al. conducted transcriptomic analyses to understand the cellular and molecular mechanisms underlying SM-induced corneal injury (Kumar et al., 2023a). Using RNA-seq profiling, the study identified 5,930 differentially expressed genes in SM-damaged corneas compared to naïve corneas, with alterations in pathways related to apoptosis, cell adhesion, migration, differentiation, proliferation, ECM remodeling, and tumor necrosis factor signaling. Protein–protein interaction (PPI) network analysis highlighted key molecular regulators, including MMPs (MMP-9, MMP-11), proinflammatory cytokines (IL-1β, TNF-α), and cell differentiation markers (CCR7, CSF1R, ITGAM, and ITGAX), which are critical in corneal fibrosis and wound healing. The study revealed that SM exposure disrupts corneal epithelial–stromal integrity, promotes keratocyte apoptosis, induces fibroblast-to-myofibroblast transition via Smad2/3 signaling, and upregulates inflammatory mediators, contributing to progressive corneal haze and fibrosis. These findings provide new insights into the molecular pathology of MGK and highlight potential therapeutic targets for mitigating SM-induced corneal damage.

In another study, exposure of the cornea and corneal keratocytes to NM led to sustained activation of the unfolded protein response (UPR) and enhanced VEGF signaling (Zhylkibayev et al., 2024). These molecular events contributed to cell death through both apoptosis and ferroptosis pathways. Subsequently, the same research group observed similar effects in corneal tissue exposed to lewisite and PAO, including the activation of pro-inflammatory cytokine signaling, indicating that these vesicants may share common downstream injury mechanisms in the eye (Zhylkibayev et al., 2023).

#### 5.3.1. Role of inflammation, oxidative stress, and cell death pathways

Inflammation is a key driver of corneal tissue damage following injury. Immune cells, such as neutrophils, macrophages, and dendritic cells, infiltrate the cornea, where they release proinflammatory cytokines and ROS. The inflammatory response is largely mediated by TNF-α, IL-1β, IL-6, and IL-8, which promote epithelial apoptosis, fibroblast proliferation, and neutrophil recruitment. Sustained inflammation leads to chronic corneal haze, persistent wound healing defects, and neovascularization, further exacerbating tissue damage (McNutt, 2023).

Oxidative stress plays a crucial role in corneal injury by generating excessive ROS, leading to lipid peroxidation, protein oxidation, and mitochondrial dysfunction. Endogenous antioxidant systems, including superoxide dismutase, catalase, and glutathione peroxidase, serve to neutralize ROS; however, their depletion during injury enhances oxidative damage. The transcription factor nuclear factor erythroid 2-related factor 2, which regulates antioxidant defenses, is often impaired in chronic corneal disease, reducing the ability of the cornea to counteract oxidative damage (Cejka and Cejkova, 2015; Hua et al., 2017; Nita and Grzybowski, 2016).

**Fig. 7.**
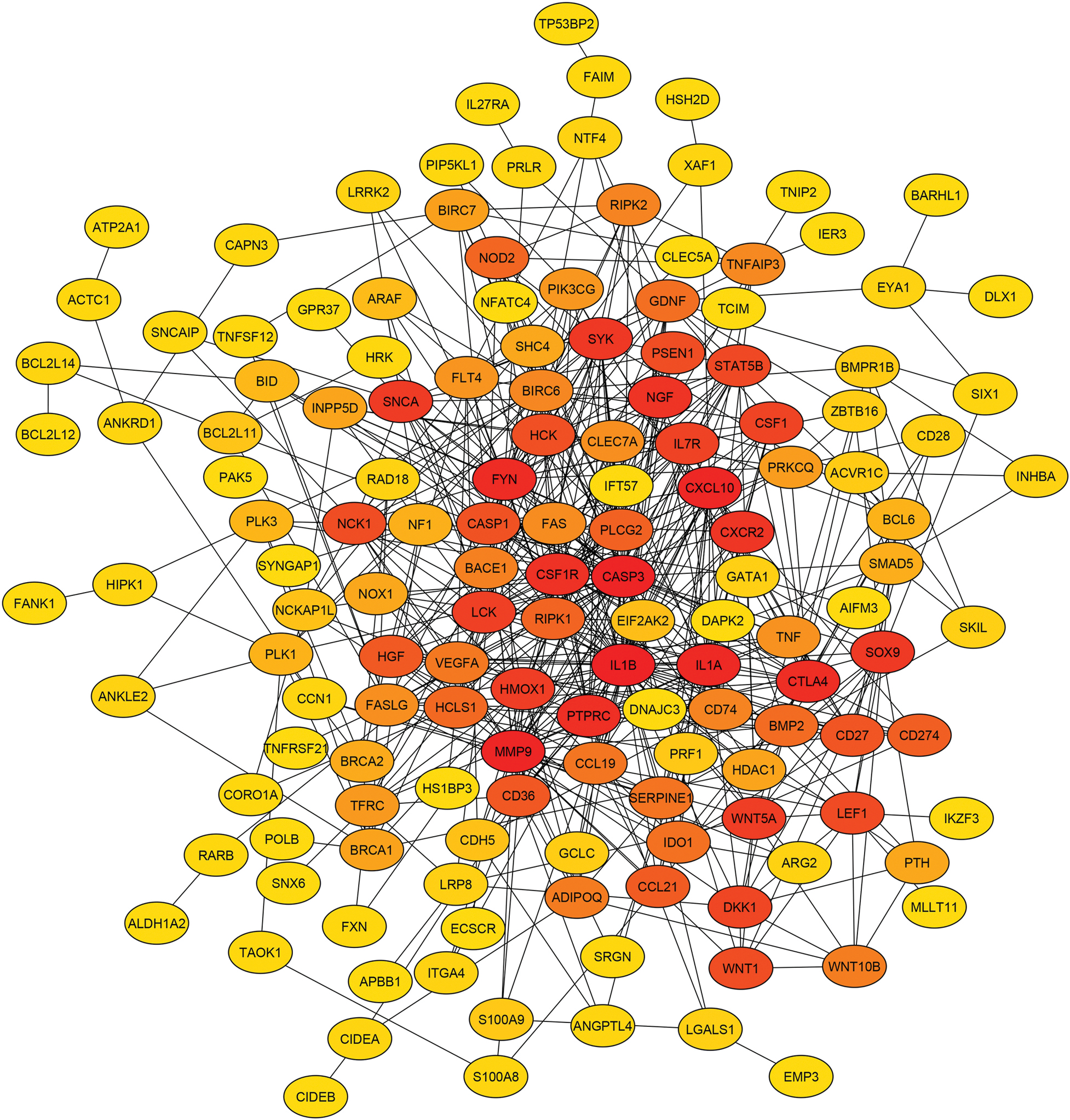
Protein-Protein Interaction network of enriched genes involved in the cell death pathway in SM-injured versus naïve rabbit corneas. The network was generated using Cytoscape and the cytoHubba plugin was used to identify the influential nodes within the network. Nodes are colored based on their influence, with red indicating highly influential node that play a critical role within the network. Edges represent interactions between proteins, that emphasize the interconnectedness of the nodes implicated in cell death processes following mustard gas exposure (adapted from Sinha et al., 2025). (For interpretation of the references to color in this figure legend, the reader is referred to the Web version of this article.)

Various forms of cell death contribute to corneal injury, including apoptosis, necroptosis, and ferroptosis. Apoptosis is a caspase-mediated process that results in controlled cell death, whereas necroptosis, a regulated form of necrosis triggered by RIPK1/RIPK3 signaling, leads to inflammatory cell lysis. Ferroptosis, characterized by iron-dependent lipid peroxidation and glutathione depletion, has recently been implicated in oxidative stress–induced corneal endothelial cell damage. These cell death pathways collectively contribute to progressive corneal degeneration, making them potential therapeutic targets for corneal repair strategies (Fig. 7) (Kumar et al., 2023a; Sinha et al., 2025b; Yan et al., 2024).

#### 5.3.2. Specific signaling pathways and molecular markers involved

**Fig. 8.**
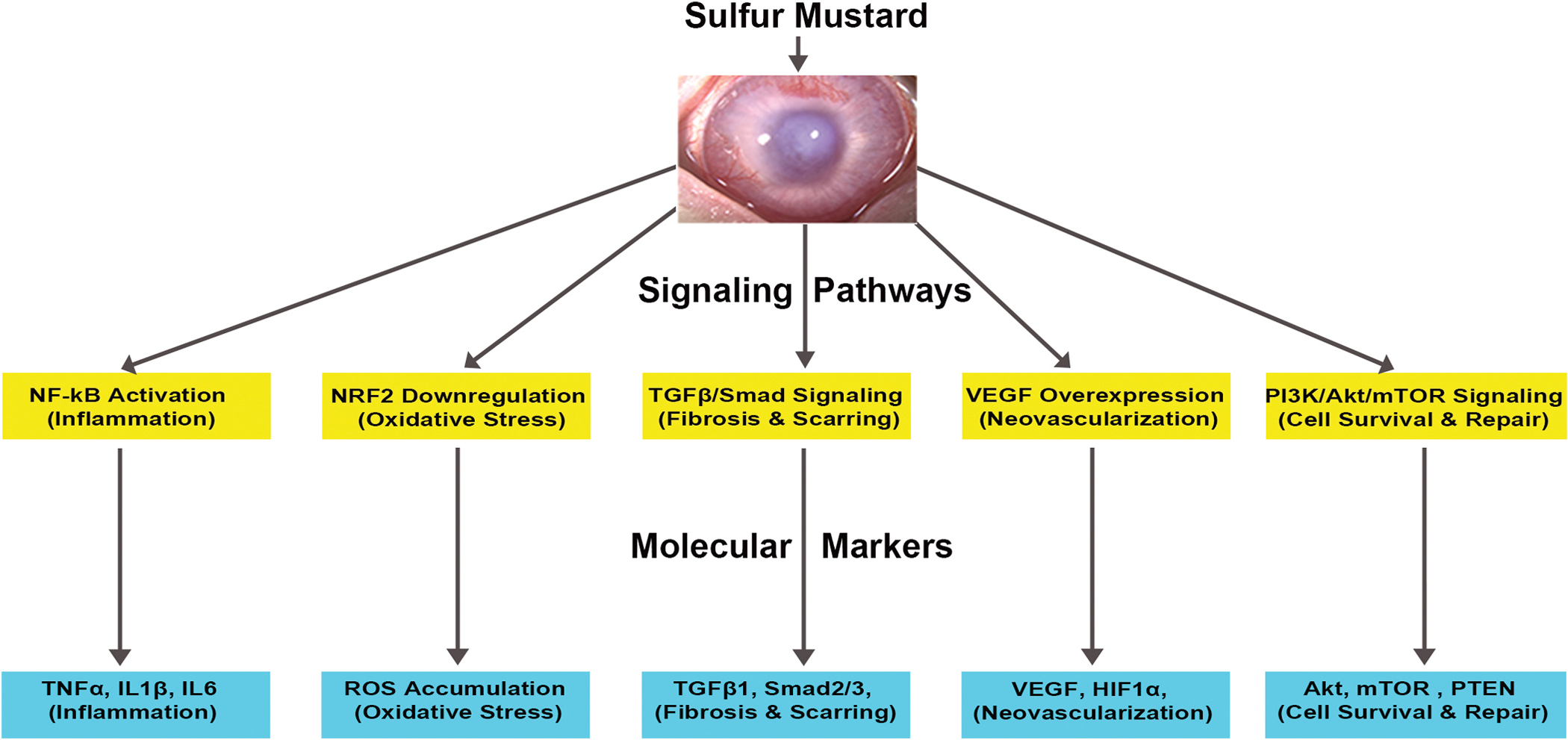
Sulfur mustard exposure to eyes induces a complex network of signaling pathways in the cornea and contributes to inflammation, oxidative stress, fibrosis, neovascularization, and repair. Key pathways include NF-κB activation, which promotes inflammation through increased expression of proinflammatory cytokines such as TNF-α, IL-1β, and IL-6; and NRF2 downregulation, which leads to the accumulation of reactive oxygen species, contributing to oxidative stress. The TGF-β/Smad signaling pathway is activated, driving fibrotic remodeling and stromal scarring via markers such as TGF-β1 and Smad2/3. Concurrently, VEGF overexpression facilitates neovascularization through the upregulation of VEGF and HIF-1α. Also, PI3K/Akt/mTOR signaling is involved in promoting cell survival and tissue repair by regulating downstream molecules including Akt, mTOR, and PTEN.

Several signaling pathways regulate corneal tissue damage and repair (Fig. 8). The TGF-β/Smad pathway plays a crucial role in fibrosis and scarring. Injury-induced upregulation of TGF-β1 promotes myofibroblast differentiation, leading to excessive ECM deposition. Activation of the Smad2/3 pathway enhances the fibroblast-to-myofibroblast transition, resulting in stromal scarring and haze. Therapeutic inhibition of TGF-β signaling, using agents such as decorin and pirfenidone has shown promise in reducing corneal fibrosis and improving healing outcomes (Sinha et al., 2023).

CNV, a major complication of chronic corneal injury, is driven primarily by VEGF signaling. The transcription factor hypoxia-inducible factor-1 alpha (HIF-1α) upregulates VEGFR-2, leading to endothelial cell proliferation and migration, which disrupts corneal transparency. Anti-VEGF therapies, such as bevacizumab, have been employed to suppress pathological angiogenesis and restore corneal avascularity (Chang et al., 2012; Roshandel et al., 2018).

Inflammatory responses in the cornea are largely mediated by the nuclear factor kappa-light-chain-enhancer of the activated B cell (NF-κB) pathway. NF-κB is activated in response to cytokines, ROS, and pathogen-associated molecular patterns (PAMPs), leading to increased TNF-α, IL-1β, and IL-6 production. Chronic NF-κB activation sustains inflammation and delays wound healing. Inhibitors such as curcumin and resveratrol have been investigated for their potential to suppress NF-κB activity and mitigate corneal inflammation (Koganti et al., 2022; Mohan et al., 1998).

The Wnt/β-catenin pathway plays a dual role in corneal regeneration and fibrosis, depending on its regulation. Controlled activation of β-catenin promotes epithelial proliferation and repair, whereas excessive signaling leads to fibroblast activation and scarring. Understanding the fine balance of Wnt signaling could offer novel approaches to improving corneal wound healing while preventing fibrosis (Mishra et al., 2021; Nakatsu et al., 2011).

Aquaporins (AQPs) are transmembrane water channel proteins that regulate corneal hydration, osmolarity, and wound-healing responses. AQP1, AQP3, and AQP5 play crucial roles in epithelial cell migration, proliferation, and ECM remodeling following corneal injury. Dysregulation of AQPs, particularly due to chemical injuries such as SM exposure, leads to corneal edema, inflammatory responses, and impaired transparency. Downregulation of AQP1 and AQP5 postinjury is associated with delayed corneal healing, while AQP3 deficiency disrupts glycerol transport, impairing epithelial repair (Bhend et al., 2023). Targeting AQPs to restore their physiological expression and function presents a potential therapeutic strategy for mitigating corneal damage and fibrosis.

The PI3K/Akt/mTOR pathway is essential for cell survival, proliferation, and migration in response to corneal injury. Akt activation promotes epithelial and endothelial cell repair mechanisms, enhancing regenerative capacity. However, chronic mTOR signaling dysregulation contributes to fibrotic changes, highlighting the need for selective therapeutic modulation of this pathway to optimize corneal healing (Chen et al., 2022; Mishra et al., 2021).

These pathway-specific insights describe the complex molecular crosstalk that determines whether corneal injury resolves with transparent regeneration or progresses to chronic opacity and vision loss. The recurring convergence of profibrotic (TGF-β/Smad, Wnt/β-catenin), proangiogenic (VEGF/HIF-1α), proinflammatory (NF-κB), and dysregulated survival (PI3K/Akt/mTOR) signals suggests that corneal pathology is not driven by a single axis and involves multiple synergistic/sequential cascades. Vesicants such as SM appear to exacerbate corneal homeostatic imbalance by simultaneously triggering fibrotic, angiogenic, and inflammatory programs while impairing vital mechanisms such as aquaporin-mediated hydration control. These mechanistic insights argue for the development of multi-modal interventions and MCMs employing TGF-β, VEGF, and NF-κB inhibitors and modulators of epithelial regeneration to reverse corneal damage and resulting vision loss. Moreover, precise timing of pathway modulation, informed by stage-specific biomarker profiling, may be critical to preserve regenerative potential while preventing fibrosis and neovascularization in tailored interventions for SM-induced corneal injury.

## 6. Pathobiology of retinal injury following exposure to vesicants

### 6.1. Retinal phenotype as a result of exposure to vesicants

Research investigating the effects of vesicating agents on retinal injury has been quite contradictory until recently. For example, a study conducted with NM and other alkylating agents, nitroimidazole and tirapazamine, originally proposed as anticancer agents for tumor hypoxic cells, evaluated retinal damage during systemic drug administration (Lee and Wilson, 2000). In a 1999 study, Lee and Wilson observed irreversible retinal toxicity characterized by photoreceptor cell apoptosis following the application of tirapazamine and nitroimidazole in rodents and monkeys, which ultimately led to the termination of their preclinical development. However, systemic treatment of mice with 240 μg/kg of the alkylating vesicant NM did not result in immediate retinal cell loss 10 days posttreatment, supporting its potential as an anticancer drug (Lee and Wilson, 2000). In 2003, Banini et al. investigated the effect of NM on ocular health in rabbits. Topical corneal application of 1 % NM followed by the administration of saline was used to mimic direct ocular exposure to mustard on a battlefield (Banin et al., 2003). This study also reported no retinal functional loss at 6–7 weeks after NM-induced ocular injury; mixed rod–cone responses, rod responses, and light-adapted cone responses (at 1- and 30-Hz flicker) did not show a difference between NM-injured and fellow control eyes in rabbits, confirming the results of the study of NM in mice (Lee and Wilson, 2000).

The fact that retinal damage presents among other ocular symptoms in individuals exposed to vesicants for the first time has been revealed through retrospective studies of survivors of multiple large-scale SM exposures between 1983 and 1988. This exposure resulted in the deaths of 10,000 Iranian combatants and innocent civilians (Ghavami Shahri et al., 2022; Shoeibi et al., 2017). Thus, a 2016 study examining a 41-year-old Iranian war veteran reported visual loss in the patient’s left eye and nonischemic central retinal vein occlusion (Shoeibi et al., 2016). After receiving four sessions of intravitreal bevacizumab injections over the subsequent two years, the patient’s visual acuity in the left eye improved to 20/25, and macular edema resolved without the need for further intervention. In this study, the clinical researchers concluded that SM exposure may trigger the development of central retinal vein occlusion later in life (Shoeibi et al., 2016). They proposed that proliferating bone marrow cells affected by SM might have impaired repair ability, inducing long-term respiratory and ocular complications (Shoeibi et al., 2016). A separate retrospective study was conducted with 40 survivors of the Iraq–Iran war in 2017 (Shoeibi et al., 2017). Observing these individuals 40 years later, clinicians detected a significant loss of rod-originated ERG amplitude along with the 30 Hz flickering responses originated by cone photoreceptors. Moreover, not only were the ERG amplitudes in these individuals affected, but the implicit time of the responses was diminished as well, suggesting a dramatic decline in retinal function overall (Shoeibi et al., 2017). The authors concluded that SM toxicity manifests delayed toxic effects in the retina, but not in the retinal pigment epithelium layer. SM is expected to have long-term effects on neural tissues, and the eye is made up of such tissue (Shoeibi et al., 2017).

Over the past decade, studies on ocular alkali burns have revealed severe retinal damage, although originally damaged corneal tissue (Huang et al., 2022; Paschalis et al., 2017, 2018; Xie et al., 2020). These findings likely spurred interest in thoroughly investigating whether the DOE to vesicants, damaging corneal tissue, also affects the posterior pole of the eye. The Gorbatyuk’s group developed an interest in investigating arsenic-based vesicants. Thus, in 2023, they validated the detrimental effects of lewisite exposure on neural retina tissue. Using a vapor cap method to simulate DOE to lewisite, we identified significant retinal damage alongside corneal injury (Zhylkibayev et al., 2023). Their findings revealed apoptotic cell death across key retinal cells, including retinal ganglion cells (RGCs), photoreceptors, and inner nuclear layer cells. To model these effects more feasibly, they then used PAO, a vesicant arsenoxide compound known for inhibiting PP2 activity through stable interactions with protein thiol groups. PAO, widely employed in biochemistry and biological studies, serves as a less toxic analog of lewisite while accurately replicating its molecular pathogenesis. Like lewisite exposure, PAO caused pronounced retinal damage, inducing apoptotic cell death and reducing electroretinogram (ERG) amplitudes in a dose-dependent manner. Therefore, they proposed a prototype mouse model for arsenical-induced ocular damage. This model offers a valuable tool for uncovering key cellular signaling pathways involved in retinal damage pathobiology and serves as a platform for evaluating medical countermeasures against the progression of ocular injuries (Zhylkibayev et al., 2023).

A detailed study on lewisite-induced retinal degeneration was subsequently conducted by Gorbatyuk’s group focusing on retinal functional tests and cell death at both the subacute and chronic stages (Zhylkibayev et al., 2025). Their analysis revealed that lewisite causes a severe and progressive decline in photoreceptor and RGC function in exposed mice. Over the course of 14 days (subacute stage) and 40 days (chronic stage), they consistently observed significant reductions in the amplitudes of the A- and B-waves in both scotopic and photopic ERGs, as well as diminished photopic negative response (PhNR)-in photopic ERGs. These reductions in the ERG amplitude recordings correlated with a pronounced increase in TUNEL-positive cells, indicating elevated apoptosis in the retinal tissue.

**Fig. 9.**
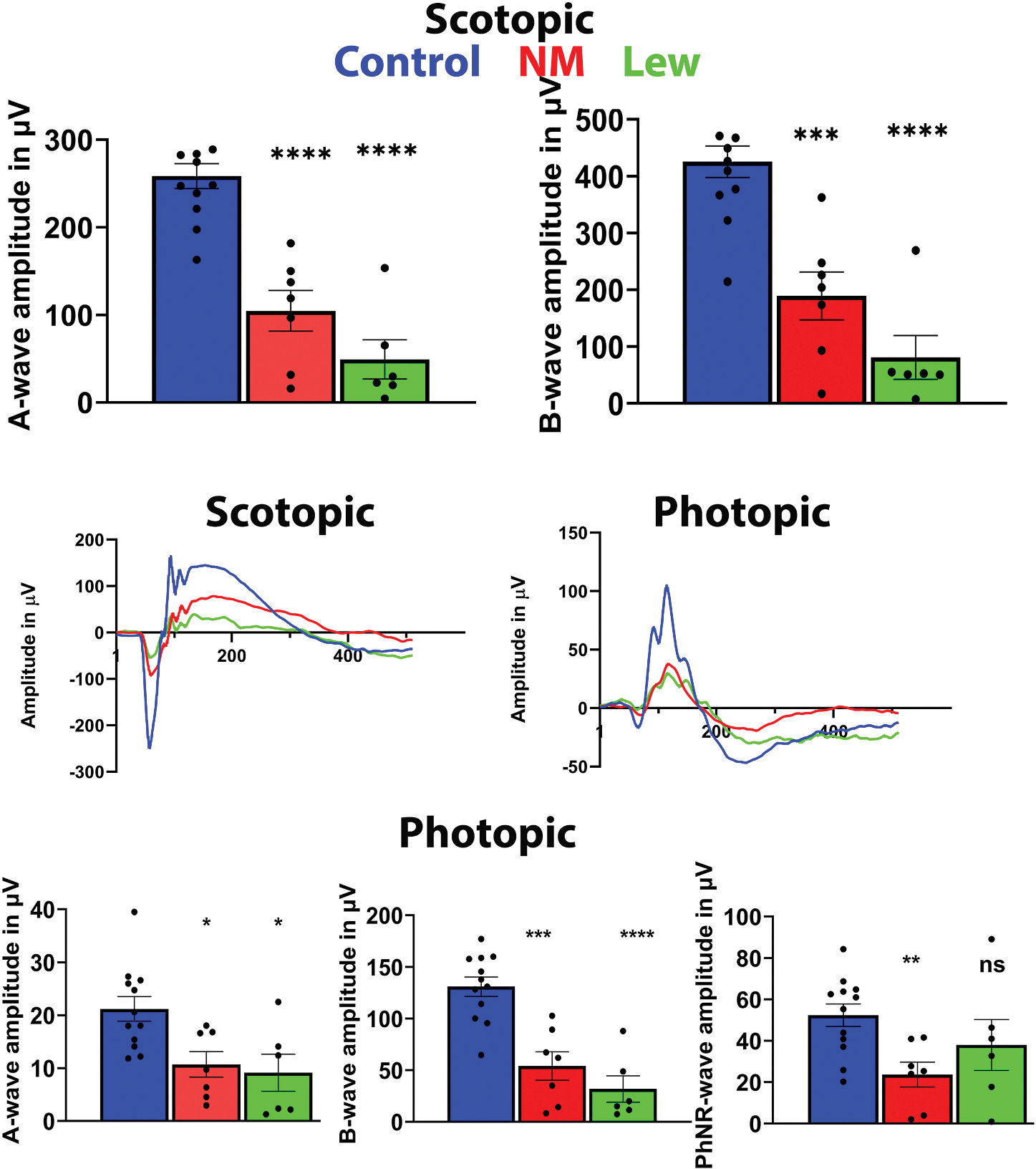
Retinal functional loss was measured by scotopic and photopic ERG in mice following direct ocular exposure to nitrogen mustard and lewisite at 40 days post exposures. Retinal degeneration was characterized by the functional loss of rods and cones, as indicated by reductions in the scotopic and photopic a-waves. Additionally, impaired function of bipolar and Müller cells was reflected in diminished scotopic and photopic b-waves. Furthermore, a decline in the photopic negative response (PhNR) provided evidence of retinal ganglion cell damage.

They then investigated whether the effects of the arsenical vesicant lewisite on retinal function differed from those induced by NM. Considering the findings by Lee et al., which demonstrated no significant retinal damage 10 days after systemic delivery of NM at a dose of 240 μg/kg of body weight (Lee and Wilson, 2000), they extended the study duration and evaluated NM’s effects through DOE. Mice whose right eye was subjected to DOE to NM were monitored over a 40-day period to assess NM impact on retinal function. The results revealed that, similar to lewisite-mediated reductions in rod- and cone-mediated ERG amplitudes, NM exposure also caused severe retinal damage, with progressive functional decline and photoreceptor cell death over the 40-day period (Zhylkibayev et al., 2024). A comparison of the effects of these two vesicants on retinal function is shown in Fig. 9. Their findings on vesicant-induced retinal injury were later confirmed by Mandal et al., who reported significant reductions in the scotopic negative rod-derived A-waves and positive bipolar-originated B-waves in NM-treated mice compared to saline-treated controls at 15 and 35 days post-exposure (Basu et al., 2024).

Clinically, retinal histopathological changes observed in mice exposed to NM and lewisite, along with documented retinal damage in individuals subjected to large-scale SM exposures between 1983 and 1988, suggest that vesicant exposure may trigger the development of retinal degeneration. This degeneration is characterized by reduced visual acuity, diminished rod-driven ERG responses, and impaired 30 Hz flicker responses originating from cone photoreceptors. The delayed onset of retinal dysfunction is likely associated with both the duration of exposure and the dose received.

### 6.2. Molecular pathogenesis driving retinal tissue damage

The molecular mechanism driving vesicant-induced retinal pathobiology remains under investigation, largely because the potential retinal damage resulting from vesicant exposure has only recently garnered significant attention. Although corneal damage following exposure to vesicants SM, NM, and lewisite has been comprehensively studied, the long-term effects of these agents on retinal homeostasis are still poorly understood. Retinal injury post-exposure could be triggered by various stimuli originating from corneal and vascular endothelial cells. Consequently, identifying molecular signals transmitted by corneal and corneal neovascularization that could initiate retinal pathogenesis represents a promising area for future research (Fig. 1). Overall, 2023 was a year of significant discoveries regarding the long-term impact of ocular vesicant exposures, with a particular focus on retinal injury phenotypes and their molecular mechanisms. In a study by Zhylkibayev et al., Gorbatyuk team reported that retinal tissues of mice with DOE to vesicant PAO exhibited a significant elevation in the mRNA expression of cytokine Il-β, Il-6, and Cox2 24 h post-exposure (Zhylkibayev et al., 2023). Furthermore, consistent with observations in corneal and retinal endothelial cells, the PAO-treated retina demonstrated sustained activation of the protein kinase R-like endoplasmic reticulum kinase (PERK) arm of the UPR. Notably, the growth-arrest-and DNA-damage-induced protein 34 (GADD34) and activating transcriptional factor 4 (ATF4) were dramatically upregulated in these retinas. These findings align with the previous studies implicating the UPR activation and inflammatory response in the pathogenesis of skin lesions and acute kidney induced by arsenical vesicants (Srivastava et al., 2016, 2020).

This year, Chaurasia and Mohan groups jointly reported retinal Müller cell damage induced by SM and NM in both *in vitro* and *in vivo* models (Mahaling et al., 2023; Umejiego et al., 2023). Müller cell hyperactivity was observed through the upregulation of glial fibrillary acidic protein (GFAP) and vimentin. *In vitro* experiments revealed that this hyperactivation was accompanied by an increased expression of caspase-1, NLRP3, IL-1β, IL-18, and elevated Gasdermin D (Mahaling et al., 2023). The authors concluded that NM-induced Müller cell gliosis occurs via heightened oxidative stress and leads to caspase-1-dependent activation of the NLRP3 inflammasome, resulting in cell death primarily driven by pyroptosis (Mahaling et al., 2023). In the corresponding *in vivo* study, reactive gliosis was also observed, suggesting that it may contribute to retinal degeneration in mice (Umejiego et al., 2023). In addition, scientists have demonstrated that 1 % NM exposure induces retinal gliosis and citrullination, as measured by fluorescence-based F95 immunoreactivity in the acute and immediate reactive phases (Umejiego et al., 2023). As a post-translational modification, citrullination converts the amino acid arginine in a protein into citrulline, altering protein biology, including its structure, stability, localization, nucleic acid binding, and catalytic activity. Moreover, in addition to proposing citrullination as a key molecular mechanism, this study revealed an increase in retinal apoptotic cell death, further highlighting the consequences of NM exposure on retinal health (Umejiego et al., 2023).

A multiomic study on the retinal tissue of mice with DOE to vesicants was conducted by Gorbatyuk’s group. Analyzing the retinal proteomics from acute stages (2 days post-exposure) and chronic stages (40 days) and comparing the chronic stages following exposure to two vesicants, NM and LEW, we identified common proteins and signaling pathways altered in the exposed mice. Thus, we learned that the top three common retinal proteins altered in NM- and lewisite treated mice were annexin A6, adenylyl cyclase-associated protein 1, and elongation factor 1-gamma. At the acute stage of post-NM exposure, they identified DNA topological changes that align with the alkylating mode of NM action. The top canonical pathways identified by ingenuity pathway analysis (IPA) include eukaryotic translation activation and termination, as well as nonsense-mediated decay. The top toxic pathways identified by IPA, including increased fatty acid metabolism and proteinuria induced by oxidative stress, ultimately resulted in a decrease in phototransduction and visual system development at 2 days post NM exposure.

Comparison of the retinal protein landscape at chronic stages following exposure to NM and lewisite revealed that both vesicants induce mitochondrial dysfunction, activation of RXR/PPAR and RXR/LXR altering actin cytoskeleton signaling. They then identified an increase in tight junctions as a common KEGG pathway activated at the chronic stage, while decreases in phototransduction and spliceosome pathways were common to both vesicant-induced retinal injuries (Zhylkibayev et al., 2025).

Retinal metabolomics was the next analysis performed on NM-exposed mice. At the chronic stage, we identified significant alterations in metabolites, including amino acids and their derivatives, fatty acids, benzenes, and glycerophospholipids, in NM-treated retinas. Notably, both reduced and oxidized glutathione (key amino acid metabolites and small peptides) were elevated 28-fold and 75-fold, respectively, indicating a pronounced state of oxidative stress. The GSSG/GSH ratio was measured at 2.6. This oxidative imbalance was further supported by a substantial increase in oxidized lipids, collectively providing evidence of oxidative stress. Further lipidomic analysis of the NM-exposed retinas revealed a significant upregulation of phosphatidylserine, a surface marker of apoptosis, and phosphatidylglycerol (20:4_20:4), an essential membrane constituent. In addition, lysophosphatidylcholine, a component of oxidized low-density lipoproteins (Ox-LDL) involved in inflammation, was markedly elevated alongside carnitines. Notably, the biogenesis and degradation of fatty acids, as well as the levels of free fatty acids, were diminished in NM-treated retinas in the chronic stages.

Lipids were also the focus of another *in vivo* study conducted by Nawajes Mandal et al. (Basu et al., 2024). Using DOE to administer 2 % NM in mice, the researchers observed an increase in aSMase activity, accompanied by a rise in Cer and HexCers and a decrease in SM during the early acute phase (three days post-exposure). These alterations indicate the synthesis of Cer from SM by aSMase. Additionally, the study reported a significant increase in cytokines and proinflammatory markers, including IL-1α, IL-1β, IL-2, TNF-α, MCP-3, VEGF, and MIP-2. These findings suggest that, along with aSMase activation and altered ceramide and SM levels, these early molecular changes could be critical events in NM-induced ocular pathology and potential targets for MCM development. Supporting this conclusion, their previously mentioned study also found increased SM, Cer, and HexCer levels in the chronic stages of NM-mediated injury, indicating a disruption in retinal lipid metabolism (Zhylkibayev et al., 2025).

What are the underlying mechanisms leading to retinal neuron cell death following vesicant exposure? The molecular mechanism of retinal cell loss due to vesicant exposure is likely multifactorial and diverse, potentially triggered not only by the direct effects of the vesicant but also indirectly through signaling molecules traveling from the cornea through the vitreous humor to the retina. For example, corneal damage and neovascularization following lewisite and SM exposure in rabbits and mice are accompanied by VEGF secretion (Goswami et al., 2021; Tewari-Singh et al., 2017a; Zhylkibayev et al., 2024). Moreover, anti-VEGF treatment with bevacizumab or eye trap (aflibercept) at 2- or 4-weeks post-exposure significantly reduces the severity of ocular injury (Gore et al., 2023; Kadar et al., 2014). The latest point suggests that secreted VEGF could the molecule traveling through the vitreous humor to the retina, inducing retinal damage. In agreement with this, Gorbatyuk’s group recently found significant upregulation of VEGF immunoreactivity in the retinas of mice with DOE to PAO, NM, and lewisite (Fig. 1). Although vesicant-induced mechanisms of cell death may be common, including oxidative stress leading to mitochondrial damage and inflammation characterized by microglial activation and cytokine release, unique retinal cell-specific responses may determine the mode of retinal cell destruction. For example, photoreceptor cells are highly sensitive to hypoxia, oxidative stress, and lipid peroxidation due to their high membrane content. In addition, RGC cells may be more susceptible to glutamate excitotoxicity triggered by Müller cells, while amacrine and bipolar cells may be more sensitive to metabolic alterations and neurotoxic stressors. These factors most likely dictate whether the dying cell proceeds with apoptosis, necroptosis, pyroptosis, or ferroptosis.

## 7. Medical countermeasures against ocular injury following exposure to vesicants

SM reactivity within biological tissues is rapid and currently irreversible (Guild et al., 1941). Consequently, treatment strategies focus on mitigating the acute injury and attenuating persistent corneal symptoms (Safarinejad et al., 2001). Given the potential for mass casualties, the lack of therapeutics, and the risk of permanent visual impairment in survivors, there is an urgent need for treatments for ocular SM injuries. Supportive treatments have shown limited ability to manage MGK sequelae, resulting in poor quality of life, while surgical interventions have mixed results, with successes limited to patients with mild symptoms of MGK (Baradaran-Rafii et al., 2013; Javadi et al., 2007, 2011; Mousavi et al., 2009; Richter et al., 2006). Indeed, ocular exposure to vesicants, such as SM and lewisite, results in severe corneal injury, inflammation, oxidative stress, and delayed wound healing, ultimately leading to vision impairment and permanent damage (Mishra et al., 2023; Tewari-Singh et al., 2017a). Vesicants exert their toxic effects through alkylation of cellular macromolecules, induction of oxidative stress, and prolonged inflammatory responses, making therapeutic intervention challenging. Current medical countermeasures focus on topical and systemic therapies that aim to neutralize toxic effects, reduce inflammation, prevent fibrosis, and promote corneal regeneration. However, the efficacy of these treatments remains variable, posing significant challenges in achieving long-term ocular recovery. Given that vesicants or signaling molecules can be delivered to the posterior pole of the eye both directly and systemically, these challenges may also extend to countermeasures against retinal injury.

### 7.1. Topical and systemic therapies

The therapeutic approach to vesicant-induced ocular injury involves both topical and systemic pharmacological interventions. Topical therapies are designed to deliver drugs directly to the cornea and conjunctiva, whereas systemic treatments target the underlying inflammatory and oxidative pathways that contribute to tissue damage. The primary strategies involve anti-inflammatory agents, antioxidants, growth factors, and wound healing promoters.

Topical pharmacological treatments play crucial roles in managing vesicant-induced ocular injuries. Corticosteroids, such as dexamethasone and prednisolone, are commonly used to suppress acute inflammation and immune cell infiltration (Hos et al., 2011; Nakao et al., 2007; Ryu et al., 2017). However, prolonged corticosteroid use poses risks such as corneal thinning, increased intraocular pressure, and secondary infections. Nonsteroidal anti-inflammatory drugs (NSAIDs), including ketorolac and bromfenac, provide symptomatic relief by inhibiting prostaglandin-mediated inflammation, but they do not address the underlying oxidative damage (Goyal et al., 2001; Lee and Himmel, 2006; Raj et al., 2022; Schechter and Trattler, 2010). Antioxidative therapies, such as N-acetylcysteine (NAC), ascorbic acid (vitamin C), and glutathione eye drops, have been explored to reduce oxidative stress and enhance cellular recovery (Cejka and Cejkova, 2015; Chen et al., 2017; Gupta et al., 2021; Riley, 1984). NAC, in particular, has shown promise in reducing vesicant-induced corneal damage by scavenging ROS and preventing oxidative injury. Additionally, growth factors and regenerative therapies, such as epidermal growth factor (EGF), fibroblast growth factor (FGF), and platelet-rich plasma (PRP) eye drops, have been studied for their ability to promote epithelial regeneration and corneal repair (Chen et al., 2020; Gurung et al., 2018; Jongkhajornpong et al., 2025; Peterson and Ceresa, 2021). Despite their potential, challenges related to drug stability, cost, and bioavailability limit their widespread clinical use. Delivering drugs to the damaged retina remains a significant hurdle, as injury may compromise effective penetration. In such cases, treatment strategies may need to rely on effective topical applications to the exposed cornea to support ocular recovery.

Systemic pharmacological treatments are often employed in cases of severe ocular injury where topical therapy alone is insufficient. Systemic corticosteroids, such as intravenous (IV) or oral methylprednisolone, are used to control systemic inflammation and prevent progressive corneal damage (Azevedo Magalhaes et al., 2020; Hill and Ivey, 1994). However, corticosteroid therapy is associated with the risks of immunosuppression, metabolic side effects, and the potential worsening of infections. For instance, prolonged or high-dose corticosteroid use can harm the retina, potentially causing central serous chorioretinopathy (CSCR), a condition in which fluid builds up under the retina and may lead to vision loss (Polak et al., 1995). This highlights the need for careful use of steroids in eye treatment.

Oral antioxidant supplements, including vitamin C, vitamin E, and alpha-lipoic acid (ALA), have been investigated for their ability to reduce oxidative stress and prevent secondary complications (Davidson et al., 2017; Li et al., 2023). While beneficial, systemic antioxidants have limited corneal penetration, reducing their overall efficacy. Immunomodulatory agents, such as cyclosporine A (CsA) and tacrolimus, are potent calcineurin inhibitors that have been explored for controlling chronic inflammatory responses (Gress et al., 2025; Kate et al., 2025; Liu et al., 2025). In particular, published studies indicate that CsA can attenuate retinal inflammation by inhibiting the formation of the HMGB-1 protein in diabetic rat models (Jaffe et al., 1998; Wang et al., 2020). However, systemic side effects, including nephrotoxicity and hypertension, restrict their long-term use.

### 7.2. Efficacy of various drugs and therapeutic agents

One of the major challenges in treatment is limited drug penetration and bioavailability. The cornea, an avascular and highly protective tissue, poses a significant barrier to drug delivery. Many hydrophilic or high-molecular-weight drugs fail to penetrate sufficiently deep layers of the cornea, thus limiting their therapeutic impact. To overcome these limitations, advanced drug delivery systems, such as liposomes, nanoparticles, and hydrogels, are being developed to increase bioavailability and achieve sustained drug release.

Another challenge is the prolonged inflammatory response and chronic complications associated with vesicant exposure. Even after initial injury resolution, ongoing inflammation often leads to corneal haze, persistent wound-healing defects, and an increased risk of secondary infections. Current therapies, such as corticosteroids and NSAIDs, offer only short-term relief and are unable to address long-term inflammatory sequelae. Combination therapies, including corticosteroids with antioxidants or regenerative factors, have shown promise but require careful balancing to avoid adverse effects, such as delayed epithelial healing or increased intraocular pressure.

Corneal fibrosis and scarring represent another major obstacle to effective treatment. Persistent TGF-β-mediated fibrosis and myofibroblast activation result in excessive ECM deposition, leading to corneal opacity and loss of vision. Antifibrotic agents, such as pirfenidone and decorin, have been shown to prevent excessive scarring while preserving corneal integrity (Balne et al., 2021; Fink et al., 2015; Gupta et al., 2021; Mohan et al., 2019, 2021). However, their clinical application remains limited due to formulation challenges and potential off-target effects. Additionally, the lack of effective neuroprotection contributes to delayed healing and persistent pain in vesicant-induced ocular injuries. Corneal nerve damage disrupts sensory and trophic support for the corneal epithelium, further impairing wound healing. Neurotrophic factors, such as nerve growth factor (NGF), have been studied for their ability to promote corneal nerve regeneration. However, their short half-life, high cost, and stability issues limit widespread clinical use (Yao et al., 2025).

## 8. Future directions and conclusions

### 8.1. Gaps in current knowledge and areas for future research

Despite substantial progress in characterizing vesicant-induced ocular injuries, several critical knowledge gaps persist. The incomplete elucidation of the molecular pathways driving chronic fibrosis, particularly the role of signaling cascades and epigenetic modifications in perpetuating tissue remodeling is among major challenges. Also, there is limited knowledge about the long-term sequelae of vesicant exposure in human patients, including the progression from acute injury to chronic conditions such as MGK. The variability in individual responses to vesicants and the absence of validated biomarkers for predicting outcomes or monitoring therapeutic efficacy further complicate clinical management.

Future research should prioritize the identification of biomarkers that enable early diagnosis and track the effectiveness of treatments. This includes the exploration of novel molecular targets, such as those involved in oxidative stress, inflammation, and fibrosis, through advanced techniques, such as single-cell RNA sequencing and proteomics. Moreover, the development of robust *in vitro* and *in vivo* models that more closely replicate human ocular physiology is critical. For example, bioengineered corneal models that integrate vascular and immune components could provide deeper insights into vesicant-induced damage and facilitate preclinical testing of therapies.

### 8.2. Potential advancements in treatment and prevention strategies

From a translational perspective, several pathological features of MGK share clinical similarities with other established corneal diseases, though with distinct etiological mechanisms. For example, recurrent epithelial erosions, chronic inflammation, and persistent epithelial defects in MGK resemble those observed in microbial keratitis (Alemi et al., 2023; Sinha et al., 2022; Vaidyanathan et al., 2019). Similarly, corneal thinning and stromal scarring, while also present in keratoconus and infectious keratitis, are observed in the chronic stages of MGK (Jafarinasab et al., 2010; Shohrati et al., 2007). Additionally, corneal neovascularization and corneal edema seen in MGK are characteristic of infectious keratitis and chronic conjunctivitis. Likewise, neurotrophic features such as reduced corneal sensitivity and poor wound healing resemble with dry eye disorder and neurotrophic keratopathy (Alemi et al., 2023; Fuchs et al., 2021; Kahale et al., 2025; Vera-Duarte et al., 2024). The damage to corneal endothelium, a known pathologic event in MGK, is a hallmark corneal clinical condition in patients with Fuchs’ endothelial dystrophy (Kadar et al., 2013; McNutt et al., 2020a). These clinical parallels suggest that mapping MGK symptomatology onto existing corneal pathologies may not only improve diagnostic accuracy but also facilitate the repurposing of established therapeutic interventions from other ocular diseases. Such comparative pathology could significantly accelerate the development of targeted treatments and improve clinical management strategies for vesicant-induced ocular injuries in both military and civilian settings.

Emerging regenerative approaches offer exciting possibilities for restoring corneal integrity and function after vesicant-induced injuries. Stem cell therapy, particularly the use of limbal stem cells, has shown potential for replenishing damaged epithelial cells and supporting stromal repair. Limbal stem cells can differentiate into corneal epithelial cells, aiding in the restoration of the epithelial barrier and promoting healing (Haddad and Faria-e-Sousa, 2014). Additionally, the anti-inflammatory and immunomodulatory properties of mesenchymal stem cells (MSCs), which can mitigate chronic inflammation and fibrosis, are being explored (De Miguel et al., 2025).

Tissue engineering is another promising avenue, leveraging advances in biomaterials to create scaffold-based systems that support corneal regeneration. For example, hydrogels embedded with growth factors or stem cells can provide a supportive microenvironment for tissue repair while reducing the need for systemic treatments (Zhang et al., 2025). Furthermore, biomimetic materials designed to mimic the structural and biomechanical properties of the cornea could enhance the effectiveness of tissue-engineered grafts (Wang et al., 2025).

Advances in drug delivery systems also hold considerable promise. NPs, MNs, and sustained-release ocular implants could improve the bioavailability of therapeutic agents, enabling targeted and prolonged delivery to the cornea (Alba-Molina et al., 2025; Coskun et al., 2025; Jiang et al., 2025; Yang et al., 2024). These technologies could be particularly valuable for delivering antioxidants, cytokine inhibitors, or gene-editing tools, such as CRISPR-Cas9, which have shown potential in preclinical studies (Mirjalili Mohanna et al., 2022; Yang et al., 2024).

### 8.3. Summary of key findings and final thoughts

In summary, vesicant exposure leads to dose-dependent damage to both corneal and retinal tissues in eye. Vesicant-induced ocular injuries involve a complex interplay of oxidative stress, inflammation, and fibrosis, leading to acute and chronic corneal damage and perhaps to retina as well. Current models—spanning *in vitro*, *ex vivo*, and *in vivo* systems—have significantly advanced our understanding of these processes, although limitations in translational relevance persist. Molecular insights have identified key pathways, such as the NF-κB, MAPK, and PI3K pathways, as potential therapeutic targets. Moreover, signaling pathways involved in corneal and retinal cell destruction are associated with mixed modes of cell death, including apoptosis, necrosis, and ferroptosis. Pharmacological interventions, including corticosteroids, NSAIDs, and antioxidants, have demonstrated efficacy in mitigating acute injuries, but challenges remain in addressing chronic outcomes, such as fibrosis and CNV.

In the future, regenerative approaches, including stem cell therapy and tissue engineering, offer transformative potential for the treatment of vesicant-induced injuries. These strategies, combined with advances in biomaterials and drug delivery systems, could enable targeted and sustained repair of corneal tissues. To achieve these goals, a multidisciplinary approach that integrates molecular research, innovative therapies, and advanced modeling techniques is essential. Continued investment in this field will not only improve clinical outcomes for those affected by vesicant exposure but also increase preparedness for chemical threats. By addressing current knowledge gaps and harnessing emerging technologies, the future of treatment and prevention for vesicant-induced ocular injuries holds great promise.

## Figures and Tables

**Table 1:** In vitro and *in vivo* models to study retinal injury caused by vesicant exposures.

Type of Model	Cell line or Species	Vesicants, dose	Mechanism of Injury	Experimental time points	Outcome Measures	References

*In vitro*	Human Muller cells (MIO-M1)	NM (HN3, Tris (2-chloroethyl) amine), 50–500 μM	Caspase 1 dependent activation of NLRP3 inflammasome activation	3; 24; 72 h	Cell death through pyroptosis	Mahaling et al. (2023)
*In vitro*	Human retinal endothelial cells (HREC)	NM (HN2, Mechlorethamine HCl), 100 μM	Unfolded protein response (UPR) activation; PERK pathway	24 h	Cell death	Zhylkibayev et al. (2024)
*In vitro*	HREC and Human corneal keratocytes (HCK)	Phenylarsine oxide (PAO), 200 nM	UPR activation; Increased PERK pathway markers, GADD34, ATF4, and p-eIF2α	24 h	Cell death	Zhylkibayev et al. (2023)
*In vivo*	C57/BL6 mice	NM; 10 μL of 2 % solution and 5min exposure	Disturbance of Retinal sphingolipid homeostasis	3; 7;14; 35 days	Diminished Retinal function detected by ERG	Basu et al. (2024)
*In vivo*	C57/BL6 mice	NM (HN2), 80μg/eye and 3 min exposure	UPR activation (PERK arm); increase in VEGF cytokine	14 days	Significant reduction in scotopic and photopic ERG amplitudes; cell death	Zhylkibayev et al. (2024)
*In vivo*	C57/BL6 mice; Ptch1+/−/SKH-1 mice	PAO, skin exposure, 150 μg; 50,25, and 10μg/eye and 3 min exposure.	UPR activation and inflammation seen as an increase in cytokines in the cornea and retina.	1; 3; 14; 28 days	Cell death	Zhylkibayev et al. (2023)
*In vivo*	C57/BL6 mice	NM (HN2); 0.2–1 %o solution and 5 min exposure	Protein hypercitrullination in both the corneal and retinal tissues.	1; 5; 14; 28 days	Retinal apoptosis and gliosis.	Umejiego et al. (2023)
*In vivo*	C57/BL6 mice	Phosgene oxime small vapor cap with 10 μl of liquid ~2.04 mg/kg); 15 and 30 s exposure	Upregulation of inflammation in cornea and retina	24hr	Cell death through ferroptosis	Okoyeocha et al. (2025)
